# Metabolic Reprogramming Associated with Ferroptosis Protection by an Indole-Based Antioxidant in Aβ(25–35)-Treated SH-SY5Y Cells

**DOI:** 10.3390/antiox15070798

**Published:** 2026-06-26

**Authors:** Mariapia Vietri, Enza Napolitano, Maria Rosaria Miranda, Carmen Marino, Simona Musella, Veronica Di Sarno, Carmine Ostacolo, Michele Manfra, Pietro Campiglia, Mario Felice Tecce, Anna Maria D’Ursi, Ornella Moltedo, Alessia Bertamino, Tania Ciaglia, Vincenzo Vestuto

**Affiliations:** 1 Department of Pharmacy, University of Salerno, Via G. Paolo II, 84084 Fisciano, Italy; mvietri@unisa.it (M.V.); enapolitano@unisa.it (E.N.); mmiranda@unisa.it (M.R.M.); cmarino@unisa.it (C.M.); smusella@unisa.it (S.M.); vdisarno@unisa.it (V.D.S.); costacolo@unisa.it (C.O.); pcampiglia@unisa.it (P.C.); tecce@unisa.it (M.F.T.); dursi@unisa.it (A.M.D.); moltedo@unisa.it (O.M.); abertamino@unisa.it (A.B.); 2 Department of Health Science, University of Basilicata, Viale dell’Ateneo Lucano 10, 85100 Potenza, Italy; michele.manfra@unibas.it

**Keywords:** ferroptosis, amyloid-β (Aβ), Alzheimer, indole nucleus, oxidative stress, metabolic reprogramming

## Abstract

Ferroptosis has emerged as a critical mechanism linking iron dysregulation, oxidative stress, and neurodegeneration in amyloid-associated pathologies. Building on our previous work, which identified compound 20 as a promising antioxidant and neuroprotective agent, the present study investigates the molecular mechanisms underlying its protective activity against amyloid-induced ferroptosis in human neuroblastoma SH-SY5Y cells exposed to Aβ(25–35). Compound 20 (3-(((4-hydroxybenzyl)(methyl)amino)methyl)-1-methyl-N-(2-(piperazin-1-yl)ethyl)-1H-indole-5-carboxamide) markedly counteracted Aβ(25–35)-induced ferroptotic damage by restoring intracellular glutathione levels, depleting the labile iron pool, and suppressing lipid peroxidation. In parallel, the compound significantly rescued mitochondrial membrane potential and attenuated endoplasmic reticulum (ER) expansion associated with ER stress, thereby preserving cellular homeostasis under oxidative challenge. These protective effects were further corroborated by real-time PCR analysis, which revealed the modulation of key genes involved in the oxidative stress response, endoplasmic reticulum stress, and inflammatory pathways. To gain a systems-level insight into these mechanisms, untargeted ^1^H-NMR metabolomic profiling was performed. This analysis confirmed the activation of antioxidant pathways and disclosed a significant modulation of energy metabolism and GABA-related pathways, both of which are closely linked to redox balance and neuronal resilience. Overall, these findings demonstrate that compound 20 drives metabolic reprogramming that orchestrates its multifactorial protective effect against Aβ(25–35)-induced ferroptosis by coordinating antioxidant defense, iron homeostasis, and ER stress mitigation.

## 1. Introduction

Amyloid-β (Aβ) aggregation is a central pathological hallmark of Alzheimer’s disease (AD) and is widely recognized as a primary trigger of downstream neurotoxic events, including oxidative stress, mitochondrial dysfunction, metabolic alterations, and neuronal death [1,2,3]. Beyond the formation of extracellular plaques, soluble Aβ oligomers are considered the most neurotoxic species, capable of disrupting cellular redox homeostasis and activating multiple cell death pathways. In this context, therapeutic strategies aimed at both preventing Aβ aggregation and mitigating its intracellular toxic consequences are considered highly promising.

**Figure 1:**
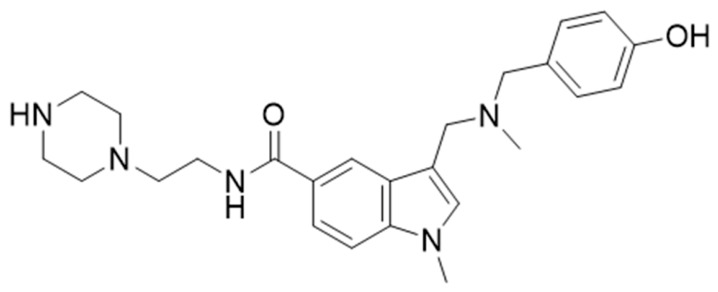
Chemical structure of compound 20 (3-(((4-hydroxybenzyl)(methyl)amino)methyl)-1-methyl-N-(2-(piperazin-1-yl)ethyl)-1H-indole-5-carboxamide).

In our previous work, we investigated the neuroprotective effects of a series of indole-based antioxidant compounds with multitarget potential against Aβ-induced neurotoxicity [4]. Among them, compound 20 (3-(((4-hydroxybenzyl)(methyl)amino)methyl)-1-methyl-N-(2-(piperazin-1-yl)ethyl)-1H-indole-5-carboxamide) (Figure 1) emerged as one of the most active molecules, displaying not only a strong antioxidant profile but also a remarkable ability to interfere with Aβ(25–35) aggregation processes. In particular, compound 20 demonstrated significant disaggregating activity against amyloid assemblies, positioning it among the most effective candidates of the investigated series. These findings suggested that the neuroprotective effects of compound 20 might arise from a combination of direct modulation of amyloid structures and downstream cellular protection.

The aforementioned study further demonstrated that compound 20 counteracted Aβ(25–35)-induced oxidative stress in neuronal cell models, restoring redox homeostasis and improving cell viability, thereby confirming a link between Aβ aggregation and oxidative damage/redox imbalance. However, accumulating evidence indicates that Aβ toxicity cannot be fully explained by generic oxidative stress alone. Instead, specific regulated forms of cell death, particularly ferroptosis, have recently gained attention as critical contributors to Aβ-induced neurodegeneration [5].

Ferroptosis is an iron-dependent, lipid peroxidation-driven form of regulated cell death, characterized by glutathione depletion, inactivation of antioxidant defenses along with lipid peroxidation, and profound metabolic remodeling [6,7]. Importantly, Aβ peptides have been shown to promote ferroptotic processes by disrupting iron homeostasis, enhancing lipid peroxidation, and impairing antioxidant systems, thereby linking amyloid aggregation, oxidative stress, and metabolic dysfunction into a combined pathogenic framework [8,9,10]. Despite this emerging evidence, the involvement of ferroptosis in the cellular response to Aβ(25–35) and its modulation by amyloid-targeting antioxidants remain poorly understood at the metabolic level [11]. Accordingly, an exhaustive metabolomic analysis of Aβ(25–35) demonstrating its capacity to replicate the ferroptosis-associated alterations commonly observed in neurodegenerative conditions has not been reported so far.

Building on our previous work, the present study aims to further characterize the biological activity of compound 20 in SH-SY5Y cells exposed to Aβ(25–35) [4], moving beyond its antioxidant and anti-aggregating properties to investigate its role in modulating amyloid-induced ferroptosis.

To this end, a comprehensive experimental approach was employed, including various biochemical assays to assess oxidative stress- and ferroptosis-related markers, real-time PCR analysis to elucidate the underlying molecular mechanisms, and an untargeted ^1^H-NMR-based metabolomic analysis to explore the global metabolic transformations and associated pathways. In this framework, NMR-based metabolomics is gaining increasing relevance due to its ability to comprehensively depict biochemical alterations at the molecular level [12,13]. Notably, recent metabolomic studies have proven effective for characterizing ferroptotic processes and investigating amyloid-related effects, and studying neuroprotective molecules targeting amyloid-induced toxicity [5,14,15,16]. By integrating molecular and metabolic data, this study seeks to provide deeper insight into the multifaceted mechanism of action of compound 20, thereby strengthening its potential as a lead compound for the development of multitarget agents against amyloid-driven neurodegeneration.

## 2. Materials and Methods

### 2.1. Cell Culture

The human neuroblastoma cell line SH-SY5Y was obtained from American Type Culture Collection (ATCC, Rockville, MD, USA). Cells were grown in Dulbecco’s Modified Eagle Medium (DMEM, 4500 mg/mL glucose) supplemented with 10% (*v*/*v*) fetal bovine serum, 2 mM L-glutamine, 100 U/mL penicillin, and 0.1 mg/mL streptomycin.

Cells were routinely grown in culture dishes (Corning, Corning, NY, USA) in a 95% humidified environment containing 5% CO_2_ at 37 °C and split every 2 days. In each experiment, cells were placed in fresh medium and cultured for 24 h alone or with compound 20 (30 µM) and Aβ(25–35) (40 µM), as reported in subsequent sections. Each treatment and analysis was performed in three separate experiments.

### 2.2. Lipid Peroxidation

Lipid peroxidation was assessed by measuring thiobarbituric acid reactive substances (TBARS), as an index of malondialdehyde (MDA) formation [17,18], using a commercial lipid peroxidation assay kit (Sigma-Aldrich, St. Louis, MO, USA). The assay is based on the colorimetric detection of MDA–thiobarbituric acid adducts, measured at 532 nm.

SH-SY5Y cells were seeded in 60 mm culture dishes at a density allowing them to reach approximately 80% confluence. After 24 h, cells were treated with compound 20 (30 µM), Aβ(25–35) (40 µM), or their combination for an additional 24 h. Untreated cells were used as negative controls, while cells treated with Aβ(25–35) alone served as positive controls for lipid peroxidation. At the end of the treatment, cells were detached, collected, and centrifuged at 655× *g* for 10 min at 4 °C. The resulting cell pellets were used to determine MDA levels according to the manufacturer’s instructions. Absorbance was measured at 532 nm using a microplate reader (Multiskan Go, Thermo Scientific, Waltham, MA, USA), and lipid peroxidation levels were expressed relative to control samples.

### 2.3. Lipid Peroxidation Assessment by C11-BODIPY Staining

Lipid peroxidation was evaluated using the fluorescent probe C11-BODIPY 581/591 (MedChemExpress, Monmouth Junction, NJ, USA)), a lipid-sensitive dye commonly used to detect lipid reactive oxygen species (lipid ROS) and ferroptosis-associated lipid oxidation [19]. Upon oxidation, C11-BODIPY exhibits a shift in fluorescence emission from red to green, with the green fluorescence selectively reflecting the oxidized form of the probe.

SH-SY5Y cells (4 × 10^4^ cells/well) were seeded in 96-well plates and allowed to adhere for 24 h. Cells were then treated with compound 20 (30 µM), Aβ(25–35) (40 µM), or their combination for an additional 24 h. Untreated cells were used as negative controls, while Aβ(25–35)-treated cells served as positive controls for lipid peroxidation.

For the pharmacological treatments with GPx4 inhibitor (GPX4-IN-3, Cat. No.: HY-141809, MedChemExpress), SH-SY5Y cells (3 × 10^4^ cells/well) were seeded in 96-well plates and allowed to adhere for 24 h. Cells were then treated with compound 20 (30 µM), GPX4-IN-3 (500 nM), or their combination for an additional 16 h. Untreated cells were used as negative controls, while GPX4-IN-3-treated cells served as positive controls for lipid peroxidation.

At the end of the treatment, cells were washed with PBS and fixed with a 3.7% formaldehyde solution for 10 min. Cells were subsequently permeabilized with 0.1% Triton X-100 (Sigma-Aldrich, St. Louis, MO, USA) at room temperature for 1 min, washed with PBS, and incubated with C11-BODIPY working solution (10 µM) prepared in serum-free medium without phenol red. Staining was carried out for 40 min at 37 °C in the dark, followed by washing to remove excess probe.

For qualitative analysis, representative fluorescence images were acquired using a ZOE™ Fluorescent Cell Imaging System (Bio-Rad, Hercules, CA, USA) at 20× magnification. For quantitative analysis, fluorescence corresponding to the oxidized (green) form of C11-BODIPY was measured using a PerkinElmer EnSpire multimode plate reader (PerkinElmer, Waltham, MA, USA), with excitation and emission wavelengths set at 500 nm and 510 nm, respectively. Fluorescence values were expressed as arbitrary units (a.u.) and normalized to control samples.

### 2.4. Intracellular Glutathione Detection

Intracellular reduced glutathione (GSH) levels were assessed using monochlorobimane (mBCl) [20], a cell-permeable fluorescent probe that forms a fluorescent adduct with GSH in the presence of glutathione-S-transferase (Sigma-Aldrich, product No. 69899). SH-SY5Y cells (4 × 10^4^ cells/well) were seeded in 96-well plates and, after 24 h of incubation, were treated with compound 20 (30 µM), Aβ(25–35) (40 µM), or their combination for 24 h. Untreated cells were used as negative controls, while Aβ(25–35)-treated cells served as positive controls for oxidative stress.

Following treatment, cells were washed twice with PBS and incubated with monochlorobimane working solution (60 µM) prepared in serum-free medium, according to the manufacturer’s instructions. After 30 min of incubation, cells were washed to remove excess probe. For quantitative analysis, fluorescence was measured using a PerkinElmer EnSpire multimode plate reader (PerkinElmer, Waltham, MA, USA) with excitation at approximately 394 nm and emission at approximately 490 nm, corresponding to the fluorescence of the monochlorobimane–GSH adduct. Fluorescence signals were expressed as arbitrary units (a.u.) and normalized to control samples. Representative images of monochlorobimane fluorescence were acquired using a ZOE™ Fluorescent Cell Imaging System (Bio-Rad, Hercules, CA, USA) at 20× magnification to assess intracellular GSH distribution qualitatively.

### 2.5. Intracellular Labile Iron Detection

Intracellular labile ferrous iron (Fe^2+^) levels were assessed using FerroOrange [21], a selective fluorescent probe for Fe^2+^ detection (Merck, Darmstadt, Germany), following the manufacturer’s instructions. SH-SY5Y cells (4 × 10^4^ cells/well) were grown in 96-well plates and allowed to adhere for 24 h. Cells were treated with compound 20 (30 µM), Aβ(25–35) (40 µM), or their combination for an additional 24 h. Untreated cells were used as controls, while Aβ (25–35)-treated cells served as positive controls for iron dysregulation. After treatment, cells were washed with PBS and incubated with FerroOrange working solution (1 µM) prepared in serum-free medium for 30 min. Excess dye was removed by washing with PBS. For qualitative analysis, representative fluorescence images were acquired using a ZOE™ Fluorescent Cell Imaging System (Bio-Rad, Hercules, CA, USA) at 20× magnification. For quantitative analysis, fluorescence intensity was measured using a PerkinElmer EnSpire multimode plate reader (PerkinElmer, Waltham, MA, USA), with excitation and emission wavelengths set at 543 nm and 580 nm, respectively. Fluorescence values were expressed as arbitrary units (a.u.) and normalized to control samples.

### 2.6. Mitochondrial Membrane Potential Determination

Mitochondrial membrane potential was measured using tetramethylrhodamine ethyl ester (TMRE) [22] (Invitrogen, Burlington, ON, Canada). After growing SH-SY5Y cells (4 × 10^4^ cells/well), the cells were treated with compound 20 (30 µM), Aβ(25–35) (40 µM), or their combination for 24 h. The cells were then washed with PBS and incubated with 100 nM TMRE in serum-free medium without phenol red for 30 min at 37 °C in the dark. Excess dye was then removed by washing with PBS, and fluorescence was measured using a PerkinElmer EnSpire multimode plate reader (excitation/emission 549 nm/574 nm). The quantitative analysis is reported as arbitrary units (a.u.) and normalized to control samples. The fluorescence representative images of live cells were captured using a ZOE™ Fluorescent Cell Imaging System (Bio-Rad, Hercules, CA, USA) at 20× magnification.

### 2.7. Endoplasmic Reticulum Expansion Evaluation

The intensity of endoplasmic reticulum (ER)-specific fluorescence, indicating ER expansion, which is characteristic of activated endoplasmic reticulum stress, was measured using the ER-ID Red analysis kit (Enzo Life Science, Farmingdale, NY, USA) [23]. Briefly, SH-SY5Y (4 × 10^4^ cells/well) were plated into 96-well plates, then compound 20 (30 µM) was added for 24 h alone or together with Aβ(25–35). Aβ(25–35) (40 µM) alone served as the positive control.

After incubation, 100 µL of 1X assay buffer with 1 µL of ER-ID Red detection reagent and 1 µL of Hoechst 33,342 nuclear dye was added to each well, and the cells were incubated for 20 min at 37 °C. The stained cells were washed, and representative images were acquired using a ZOE Fluorescent Cell Imaging System (Magnification, 20×). The fluorescence signals (ER-ID: excitation/emission 560 nm/630 nm) were read using a PerkinElmer EnSpire multimode plate reader. Fluorescence values were expressed as arbitrary units (a.u.) and normalized to control samples.

### 2.8. RNA Extraction, Reverse Transcription, and Real-Time PCR

Total RNA was isolated from treated cells after 24 h using Trizol reagent (Gibco, Thermo Fisher Scientific), according to the manufacturer’s instructions. Aliquots of total RNA for the real-time PCR test were subjected to DNase I digestion (Thermo Fisher Scientific) and reverse transcribed using M-MLV Reverse Transcriptase (Thermo Fisher Scientific) according to the manufacturer’s protocol. Thermal conditions for reverse transcription were 25 °C for 10 min, 37 °C for 50 min, and 75 °C for 15 min. In the last step, RNase H was added.

Real-time PCR was performed with LightCycler^®^ 480 System (La Roche Ltd., Basel, Switzerland) using SYBR Green detection in a total volume of 20 μL with 1 μL of forward and reverse primers (5 μM) and 10 μL of PowerUp™ SYBR™ Green Master Mix (Thermo Fisher Scientific). Values were determined from a standard curve generated from serial cDNA dilutions and normalized to GAPDH.

The primers used for the real-time PCR reactions are listed in the table below (Table 1).

The 2^−ΔΔCT^ method was used to analyze the results, and relative mRNA expression levels were determined as fold-induction relative to Ctrl cells, set as 1. Gene expression data were analyzed using two-way ANOVA, considering gene and treatment as independent variables, followed by Sidak’s multiple comparisons test. Data are expressed as fold change relative to control [24].

### 2.9. Cell Viability Assay

Cell viability was established by measuring mitochondrial metabolic activity with 3-(4,5-dimethylthiazol-2-yl)-2,5-diphenyl-2H-tetrazolium bromide (MTT) [25]. Briefly, SH-SY5Y (3 × 10^4^ cells/well) were plated into 96-well plates, then compound 20 (30 µM), GPx4 inhibitor (GPX4-IN-3, 0.5 µM. Cat. No.: HY-141809, MedChemExpress), or their combination was added for 24 h. Ferrostatin-1 (2 µM. Cat. No.: SML0583, Sigma Aldrich) was used as a positive control. Afterward, MTT reagent at 0.5 mg/mL final concentration for 1 h was added. Then, 100 μL per well of 0.1 M isopropanol/HCl solution was added to dissolve the formazan crystals. The absorbance was measured at 570 nm, using a microplate reader (Multiskan Go, Thermo Scientific, Waltham, MA, USA). Cell viability was expressed as a percentage relative to the untreated cells cultured in medium with 0.1% DMSO and set to 100%, whereas 10% DMSO was used as a positive control and set to 0% of viability.

### 2.10. Glutathione Peroxidase (GPx) Activity

GPx-related activity was assessed by measuring the consumption of reduced glutathione (GSH) in the presence of hydrogen peroxide, followed by the quantification of the residual GSH using 5,5′-dithiobis-(2-nitrobenzoic acid) (DTNB, Ellman’s reagent), with minor modifications from previously described methods [26]. SH-SY5Y cells were seeded in 6-well plates at a density of 8 × 10^5^ cells/well. Cells were treated with Aβ(25–35) and compound 20 for 24 h, according to the conditions adopted for the other experiments, whereas treatment with GPX4-IN-3 (0.5 µM) was performed for 16 h. The reaction mixture consisted of 20 µL of cell lysate (100 µg of total protein), 10 µL of 10 mM reduced glutathione (GSH), 10 µL of 10 mM sodium azide, 10 µL of 500 mM Tris-HCl buffer (pH 8.5), 8 µL of 5 mM hydrogen peroxide, and distilled water to a reaction volume of 32 µL. Subsequently, 10 µL of 30 mM DTNB was added to obtain a final volume of 100 µL, and the absorbance was measured at 412 nm using a microplate reader. Since the absorbance at 412 nm is directly proportional to the amount of residual reduced glutathione, lower absorbance values indicate increased GSH consumption and, consequently, higher GPx-related activity. The reaction blank, containing reduced glutathione in the absence of cell lysate, was set as 0% GPx-related activity, and the results were expressed as GPx-related activity (%), calculated from the relative decrease in residual GSH with respect to the reaction blank.

### 2.11. NMR Metabolomics

#### 2.11.1. Sample Preparation for Metabolomic Analysis

SH-SY5Y cells (3.5 × 10^6^ cells) were seeded in 100 mm culture dishes. After 24 h, cells were treated with compound 20 (30 µM), Aβ(25–35) (40 µM), or their combination for an additional 24 h. Untreated cells served as the control. All experimental conditions were performed in triplicate.

The growth medium and cell extracts were analyzed as previously described [27,28]. Briefly, at the end of treatment, the growth medium from each plate was collected for exometabolome analysis. It was centrifuged at 1000× *g* for 10 min to remove debris, and the supernatants were stored at −80 °C until NMR analysis. After removing the media, the dishes were washed twice with cold PBS (pH 7.4) to remove residual medium, and cells were scraped into methanol for endometabolome analysis. Intracellular metabolites were extracted by homogenization and biphasic extraction using a mixture of methanol, chloroform, and water in equal parts. The samples were centrifuged at 1600× *g* for 30 min at 4 °C to separate the polar and non-polar phases. The polar phase was dried using a SP-Genevac EZ-2 4.0 concentrator and stored at −80 °C until NMR analysis.

Lyophilized cell extracts were resuspended in 200 μL of 50 mM Na_2_HPO_4_ buffer in a 9:1 mixture of H_2_O and D_2_O, containing 1 mM trimethylsilyl propionic-2,2,3,3-d_4_ acid sodium salt (TSP-d_4_) as an internal standard. For media analysis, 100 μL of culture medium was combined with 100 μL of the same buffer, and the mixture was transferred into 3 mm NMR tubes for ^1^H-NMR experiments.

#### 2.11.2. NMR Spectra Acquisition and Processing

^1^H-NMR experiments were conducted on a Bruker Ascend™ 600 MHz spectrometer equipped with a 5 mm triple-resonance Z-gradient TXI probe (Bruker Co., Rheinstetten, Germany) at 298 K. All experiments, performed as 1D-Nuclear Overhauser Enhancement Spectroscopy (1D-NOESY), were acquired in triplicate. Spectra were collected with a spectral width of 12 ppm, 20 k data points, and 128 scans, using a 5 s relaxation delay and a 10 ms mixing time. Spectrometer control and data processing were carried out using TopSpin version 3.2 (Bruker Biospin, Rheinstetten, Germany). Spectra were analyzed using an untargeted metabolomic approach, in which each metabolite was identified prior to statistical analysis using Chenomx NMR-Suite v10.1 (Chenomx NMR Suite, v10.1, Edmonton, AB, Canada). Metabolite quantification from the 1D-NOESY spectra was performed with NMRProcFlow ver. 1.4.10 [29], and the generated data matrix was then analyzed statistically.

### 2.12. Statistical Analysis

The data are reported as mean ± SD of the results from three independent experiments. Statistical analysis was performed using an analysis of variance (ANOVA), and multiple comparisons were performed with the Bonferroni test in GraphPad Prism 8.0 (San Diego, CA, USA). Significance was assumed at *p* < 0.05.

For the metabolomic analysis, sample data from ^1^H-NMR spectra were normalized using sum normalization and Pareto scaling, then analyzed with Metaboanalyst 6.0 [30]. Univariate analysis was conducted on both the exometabolome and endometabolome using a *t*-Test and Fold Change (FC Threshold: 1); results are shown in a Volcano plot [31]. Subsequently, multivariate analysis was performed using Partial Least Squares Discriminant Analysis (PLS-DA). The reliability of the supervised model was evaluated using a 10-fold cross-validation, with accuracy and the Q2 and R2 parameters. Loading plots from PLS-DA helped identify the most influential metabolites in group separation, ranked by their Variable Importance in Projection (VIP) scores, with a significance threshold set above 1. Pathway analysis was conducted using MetPa within MetaboAnalyst 6.0. KEGG pathways were chosen based on a lower false discovery rate (FDR), *p* < 0.05, and at least two identified metabolites per pathway (hits).

Pathways were also evaluated using the Pathway Impact parameter (PI). PI combines centrality and pathway enrichment results. It is calculated by summing the importance measures of the matched metabolites and dividing by the sum of all metabolites’ importance measures in each pathway. Pathways with an impact value closer to 1 are those that most discriminate against the analyzed clusters [32].

## 3. Results

### 3.1. Compound 20 Counteracts Aβ(25–35)-Induced Ferroptotic Markers in SH-SY5Y Cells

**Figure 2:**
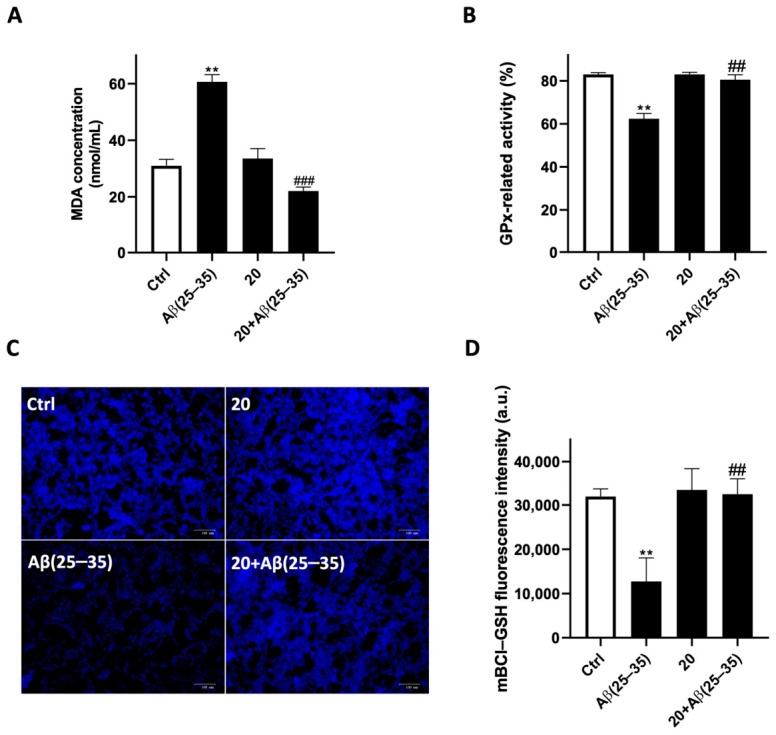
Compound 20 attenuates Aβ(25–35)-induced ferroptotic hallmarks in SH-SY5Y cells. (**A**) Lipid peroxidation end-products were assessed by measuring malondialdehyde (MDA) levels using the TBARS assay. (**B**) Determination of GPx activity against Aβ(25–35)-treated cells evaluated via spectrophotometric assay. (**C**) Representative images showing intracellular reduced glutathione (GSH) levels detected with monochlorobimane (mBCl). (**D**) Quantitative analysis of GSH in mBCl-stained cells. Scale bar: 100 μm. *n* ≥ 5. Magnification 20×. Data are expressed as mean ± SD of three independent experiments performed in triplicate. ** denotes *p* < 0.01 vs. Ctrl; ##, ### denote respectively *p* < 0.01 and *p* < 0.001 vs. Aβ(25–35).

First, lipid peroxidation was assessed by measuring malondialdehyde (MDA) levels using the TBARS assay. As expected, treatment with Aβ(25–35) caused a marked increase in MDA content (60.62 ± 2.61 nmol/mL) compared to control cells (30.95 ± 2.26 nmol/mL, *p* < 0.01 vs. Ctrl), confirming the strong pro-oxidant effect of amyloid exposure. Co-treatment with compound 20 significantly reduced MDA levels (22.01 ± 1.42 nmol/mL) compared with Aβ(25–35) alone (*p* < 0.001 vs. Aβ(25–35)), restoring lipid peroxidation levels close to basal values. Treatment with compound 20 alone did not significantly affect MDA production (33.49 ± 3.54 nmol/mL) relative to controls, indicating that the compound does not induce lipid peroxidation under basal conditions (Figure 2A).

To further investigate ferroptosis-associated redox defense mechanisms, GPx4 enzymatic activity was evaluated. Aβ(25–35) exposure significantly reduced GPx activity compared to control cells (% enzymatic activity Ctrl: 82.97 ± 0.87; % enzymatic activity Aβ(25–35): 62.39 ± 3.40, *p* < 0.01 vs. Ctrl), consistent with the impairment of cellular antioxidant capacity. Remarkably, co-treatment with compound 20 significantly restored GPx activity relative to amyloid-treated cells (% enzymatic activity 20+Aβ(25–35): 80.50 ± 2.33, *p* < 0.01 vs. Aβ(25–35)), indicating the preservation of GPx-mediated detoxification function. Importantly, compound 20 alone did not affect GPx activity, maintaining levels comparable to control cells and significantly higher than those observed upon Aβ(25–35)-exposure, further confirming that the compound does not perturb basal enzymatic function (Figure 2B).

In parallel, intracellular reduced glutathione levels (GSH) were evaluated using the monochlorobimane (mBCl) fluorimetric assay. Exposure to Aβ(25–35) resulted in a significant decrease in mBCl fluorescence intensity compared to control cells (a.u. Ctrl: 31,930.58 ± 1861.86; a.u. Aβ(25–35): 12,736.01 ± 5315.94, *p* < 0.01 vs. Ctrl), indicating depletion of the intracellular GSH pool. Notably, co-administration of compound 20 markedly restored mBCl fluorescence intensity relative to cells treated with Aβ (25–35) (a.u. 20+Aβ(25–35): 32,609.13 ± 3494.22; *p* < 0.01 vs. Aβ (25–35)), suggesting recovery of intracellular GSH levels and redox homeostasis. Treatment with compound 20 alone produced fluorescence levels comparable to those observed in the co-treatment condition and did not differ significantly from control values (Figure 2D).

Fluorescence microscopy analysis supported these quantitative findings. Representative images revealed a pronounced reduction in intracellular mBCl fluorescence in cells treated with Aβ(25–35), whereas cells co-treated with compound 20 displayed a more intense and homogeneous fluorescence signal, similar to control cells, further demonstrating the preservation of intracellular GSH distribution (Figure 2C).

Taken together, these results demonstrate that Aβ(25–35) induces a pronounced oxidative imbalance characterized by increased lipid peroxidation and depletion of intracellular glutathione. The ability of compound 20 to simultaneously suppress MDA formation and restore GSH levels suggests an upstream modulation of oxidative stress.

**Figure 3:**
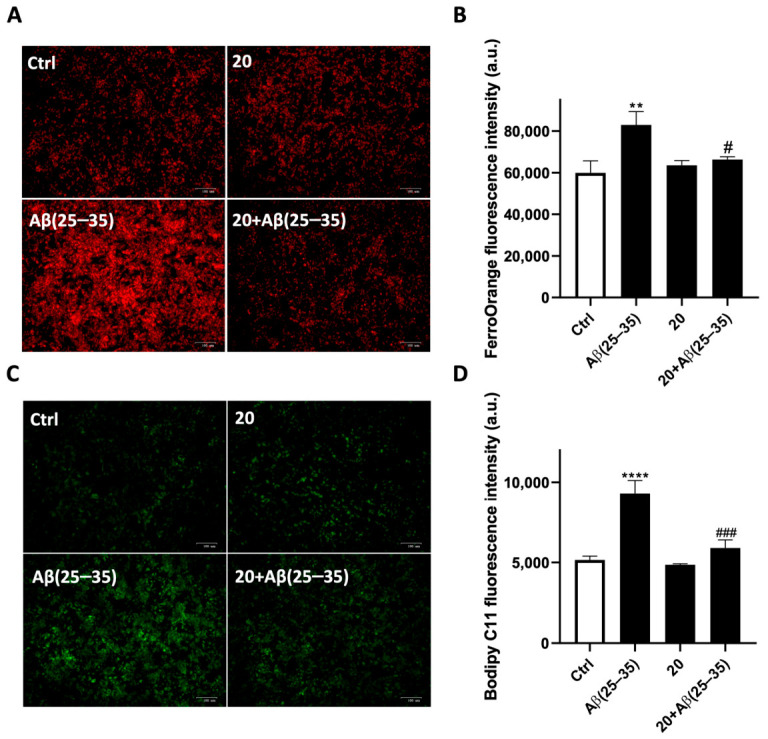
Compound 20 attenuates Aβ(25–35)-induced intracellular labile iron accumulation and lipid peroxidation in SH-SY5Y cells. (**A**) Representative fluorescence microscopy images of SH-SY5Y cells stained with FerroOrange. (**B**) Quantitative analysis of FerroOrange fluorescence intensity. (**C**) Representative fluorescence microscopy images of SH-SY5Y cells stained with BODIPY C11, showing lipid peroxidation. (**D**) Quantitative analysis of oxidized BODIPY C11 fluorescence. Scale bar: 100 μm. *n* ≥ 5. Magnification 20×. Data are expressed as mean ± SD of three independent experiments performed in triplicate. **, **** denote *p* < 0.01 and *p* < 0.0001 vs. Ctrl; #, ### denote respectively *p* < 0.05 and *p* < 0.001 vs. Aβ(25–35).

Given the close interplay between ferroptosis activation and iron overload, intracellular levels of labile ferrous iron (Fe^2+^) were next evaluated using the FerroOrange fluorescent probe. Representative images showed a pronounced increase in FerroOrange signal in cells treated with Aβ(25–35), whereas cells co-treated with compound 20 displayed a markedly reduced and more homogeneous fluorescence pattern, comparable to control cells (Figure 3A). Quantitative spectrofluorometric analysis showed that exposure to Aβ(25–35) resulted in a significant increase in FerroOrange fluorescence intensity compared to control cells (a.u. Ctrl: 59,888.33 ± 5783.49; a.u. Aβ(25–35): 82,952.67 ± 6425.72, *p* < 0.01 vs. Ctrl), indicating the accumulation of intracellular labile Fe^2+^ and iron dyshomeostasis. In contrast, co-treatment with compound 20 tended to reduce FerroOrange fluorescence relative to Aβ(25–35) treated cells (a.u. 20+Aβ(25–35): 66,299.33 ± 1361.54, *p* < 0.01 vs. Aβ(25–35)), restoring Fe^2+^ levels toward those observed under control conditions. Treatment with compound 20 alone did not significantly alter FerroOrange fluorescence compared to control cells (a.u. 20: 63,505.33 ± 2334.92), suggesting that the compound does not perturb iron homeostasis in the absence of amyloid-induced stress (Figure 3B). To determine whether iron accumulation translated into enhanced lipid oxidative damage, lipid peroxidation was further assessed using the lipid-sensitive probe BODIPY C11. Quantitative fluorescence analysis of the oxidized (green) form of BODIPY C11 revealed a significant increase in lipid peroxidation in cells treated with Aβ(25–35) compared to control cells (a.u. Ctrl: 5158.33 ± 227.15; a.u. Aβ(25–35): 9308.50 ± 810.13, *p* < 0.0001 vs. Ctrl). Notably, co-treatment with compound 20 markedly reduced BODIPY C11 fluorescence relative to cells treated with Aβ(25–35) alone (a.u. 20+Aβ(25–35): 5888.83 ± 502.10, *p* < 0.001 vs. Aβ(25–35)), indicating a clear attenuation of iron-driven lipid peroxidation. Treatment with compound 20 alone did not significantly affect BODIPY C11 fluorescence (a.u. 20: 4857.83 ± 57.18) (Figure 3D). Fluorescence microscopy images confirmed these results, showing a strong increase in oxidized BODIPY C11 signal upon Aβ(25–35) exposure, while co-treatment with compound 20 resulted in a striking reduction in lipid ROS levels, maintaining a fluorescence pattern comparable to control conditions (Figure 3C). Together with the observed increase in malondialdehyde formation and depletion of intracellular glutathione, these results indicate that Aβ(25–35) promotes a ferroptosis-like oxidative cascade characterized by iron accumulation and lipid peroxidation. The ability of compound 20 to counteract both events confirms its protective role against iron-mediated oxidative damage.

### 3.2. Compound 20 Mitigates Aβ(25–35)-Induced Mitochondrial Dysfunction and Endoplasmic Reticulum Stress in SH-SY5Y Cells

**Figure 4:**
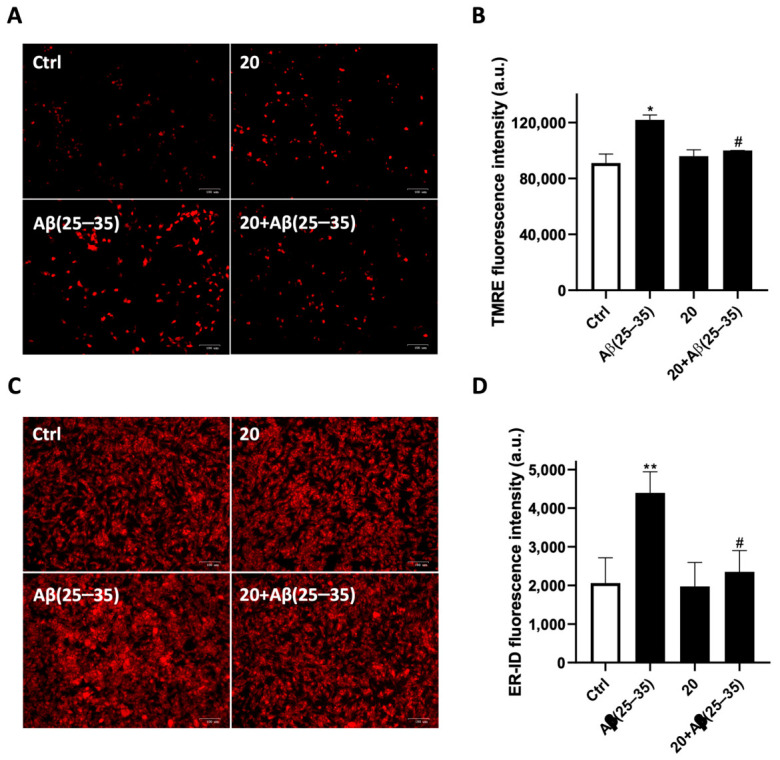
Compound 20 attenuates Aβ(25–35)-induced mitochondrial dysfunction and endoplasmic reticulum stress in SH-SY5Y cells. (**A**) Representative fluorescence microscopy images of TMRE-stained cells. (**B**) Quantitative analysis of TMRE fluorescence intensity to detect mitochondrial membrane potential. (**C**) Representative fluorescence microscopy images of ER-ID-stained cells. (**D**) Quantitative analysis of ER-ID fluorescence intensity to detect ER stress. Scale bar: 100 μm. *n* ≥ 5. Magnification 20×. Data are expressed as mean ± SD of three independent experiments performed in triplicate. *, ** denote *p* < 0.05 and *p* < 0.01 vs. Ctrl; # denotes *p* < 0.05 vs. Aβ(25–35).

To further investigate the impact of Aβ(25–35)-induced ferroptosis on organelle homeostasis, mitochondrial and ER function were subsequently examined. Mitochondrial membrane potential was assessed using TMRE staining. Quantitative fluorimetric analysis revealed that exposure to Aβ(25–35) increased TMRE fluorescence intensity compared to control cells (a.u. Ctrl: 91,001.75 ± 6438.56; a.u. Aβ(25–35): 121,926.00 ± 3471.19, *p* < 0.05 vs. Ctrl), indicating an alteration of mitochondrial membrane potential consistent with mitochondrial stress and dysfunctional polarization rather than improved bioenergetic activity(Figure 4B). In line with this interpretation, fluorescence microscopy showed a more intense and diffuse TMRE signal in cells treated with Aβ, suggesting loss of physiological mitochondrial membrane potential regulation (Figure 4A). Notably, co-treatment with compound 20 reduced TMRE fluorescence compared to cells treated with Aβ(25–35) (a.u. 20+Aβ(25–35): 99,952.25 ± 53.39, *p* < 0.05 vs. Aβ (25–35)), restoring signal intensity and intracellular distribution toward those observed in control cells. This effect indicates that compound 20 counteracts Aβ-induced mitochondrial dysfunction, preserving mitochondrial homeostasis under amyloid stress conditions. Treatment with compound 20 alone did not significantly affect TMRE fluorescence (a.u. 20: 95,940.00 ± 4495.08), confirming that the compound does not alter mitochondrial membrane potential under basal conditions (Figure 4B). Fluorescence microscopy analysis further corroborated these findings, revealing a widespread and disorganized TMRE signal in cells treated with Aβ(25–35), whereas cells co-treated with compound 20 displayed a more confined and homogeneous fluorescence distribution, consistent with preserved mitochondrial integrity (Figure 4A).

Given the tight functional crosstalk between mitochondria and the endoplasmic reticulum, particularly under conditions of oxidative stress, we next evaluated ER homeostasis using ER-ID staining, which reflects ER expansion and alterations in organelle structural integrity. Aβ(25–35) treatment resulted in a marked increase in ER-ID fluorescence intensity compared to control cells (a.u. Ctrl: 2062.33 ± 655.75; a.u. Aβ(25–35): 4395.17 ± 547.40, *p* < 0.01 vs. Ctrl), together with a diffuse intracellular staining pattern, indicative of ER stress and ER structural remodeling. Notably, co-treatment with compound 20 reduced ER-ID fluorescence (a.u. 20+Aβ(25–35): 2351.50 ± 550.56, *p* < 0.05 vs. Aβ(25–35)), restoring ER morphology and signal intensity toward control levels. Cells treated with compound 20 alone exhibited ER-ID fluorescence levels comparable to control cells (a.u. 20: 1974.17 ± 621.95) (Figure 4C,D).

Overall, these results indicate that Aβ(25–35) induces mitochondrial dysfunction and ER stress downstream of ferroptotic damage. The ability of compound 20 to attenuate both TMRE and ER-ID alterations supports its protective role in preserving organelle homeostasis, further reinforcing its efficacy in counteracting Aβ-induced cellular stress.

### 3.3. Protective Effects of Compound 20 on Gene Expression Profile of Aβ(25–35)-Treated SH-SY5Y Cells

**Figure 5:**
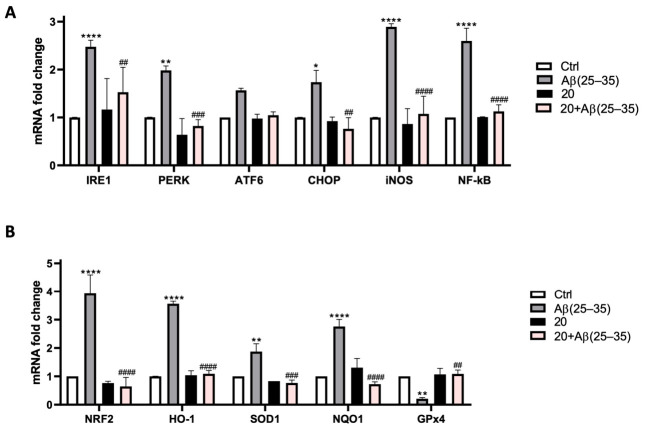
Compound 20 modulates redox, inflammatory, and ER stress-related gene expression in SH-SY5Y cells treated with Aβ(25–35). Total RNA samples extracted from SH-SY5Y cells exposed for 24 h to Aβ(25–35) were analyzed by q-PCR for (**A**) ER stress (*IRE1*, *PERK*, *ATF6*, *CHOP*), inflammatory (*iNOS*, *NF-κB*), and (**B**) oxidative stress (*NRF2*, *HO-1*, *SOD1*, *NQO1*, *GPx4*) genes. 2^−∆∆CT^ method was employed to calculate the relative quantities of mRNA. Results are expressed as fold change relative to untreated cells. Gene expression levels were normalized to the housekeeping gene *GAPDH* and expressed as fold change relative to control samples. Data are reported as mean ± SD. *, **, **** denote respectively *p* < 0.05, *p* < 0.01 and *p* < 0.0001 vs. Ctrl; ##, ###, #### denote respectively *p* < 0.01, *p* < 0.001, *p* < 0.0001 vs. Aβ(25–35).

To correlate the observed phenotypic data against ferroptosis protection with underlying molecular mechanisms, real-time PCR analyses were performed. These experiments are crucial to assess the expression of key genes involved in antioxidant defense and redox regulation, inflammatory signaling, as well as endoplasmic reticulum (ER) stress responses (Figure 5A,B). The analyzed antioxidant genes included nuclear factor erythroid 2-related factor 2 (*NRF2*), heme oxygenase-1 (*HO-1*), NAD(P)H quinone dehydrogenase 1 (*NQO1*), superoxide dismutase 1 (*SOD1*), and glutathione peroxidase 4 (*GPx4*). Furthermore, master regulators of inflammatory signaling, such as inducible nitric oxide synthase (*iNOS*) and nuclear factor κB (*NF-κB*), as well as the ER stress-related genes inositol-requiring enzyme 1 (*IRE1*), protein kinase RNA-like ER kinase (*PERK*), activating transcription factor 6 (*ATF6*) and C/EBP homologous protein (*CHOP*) were detected. Exposure to Aβ(25–35) resulted in a significant upregulation of *NRF2* (*p* < 0.0001 vs. Ctrl), *HO-1* (*p* < 0.0001 vs. Ctrl), *SOD1* (*p* < 0.01 vs. Ctrl), *NQO1* (*p* < 0.0001 vs. Ctrl) (Figure 5A), *iNOS* (*p* < 0.0001 vs. Ctrl), and *NF*-κB (*p* < 0.0001 vs. Ctrl) (Figure 5B), compared to control cells, indicating the activation of antioxidant and inflammatory stress-response pathways. In parallel, Aβ treatment induced a marked increase in the expression of *IRE1* (*p* < 0.0001 vs. Ctrl), *PERK* (*p* < 0.00 vs. Ctrl), and *CHOP* (*p* < 0.05 vs. Ctrl) (Figure 5B), reflecting activation of the unfolded protein response (UPR) and ER stress signaling. Conversely, *GPx4* expression was significantly reduced in Aβ-treated cells (*p* < 0.01 vs. Ctrl) (Figure 5B), confirming an impairment of glutathione-dependent antioxidant defenses and ferroptosis involvement. Notably, co-treatment with compound 20 markedly attenuated the Aβ-induced upregulation of *NRF2*, *HO-1*, *NQO1*, *SOD1*, *iNOS*, *NF-κB*, *IRE1*, *PERK*, and *CHOP*, restoring their expression levels toward those observed in control cells. In contrast, *GPx4* expression was significantly upregulated in the co-treatment group compared to Aβ(25–35) alone, indicating a recovery of glutathione-dependent antioxidant capacity.

Overall, these gene expression profiles closely mirror the phenotypic alterations observed during oxidative stress, iron dysregulation, mitochondrial dysfunction, and ER stress assessment, further supporting the ability of compound 20 to mitigate Aβ-induced ferroptosis by modulating redox homeostasis, inflammatory signaling, and UPR activation.

### 3.4. Compound 20 Rescues GPx4 Inhibition-Driven Ferroptosis

**Figure 6:**
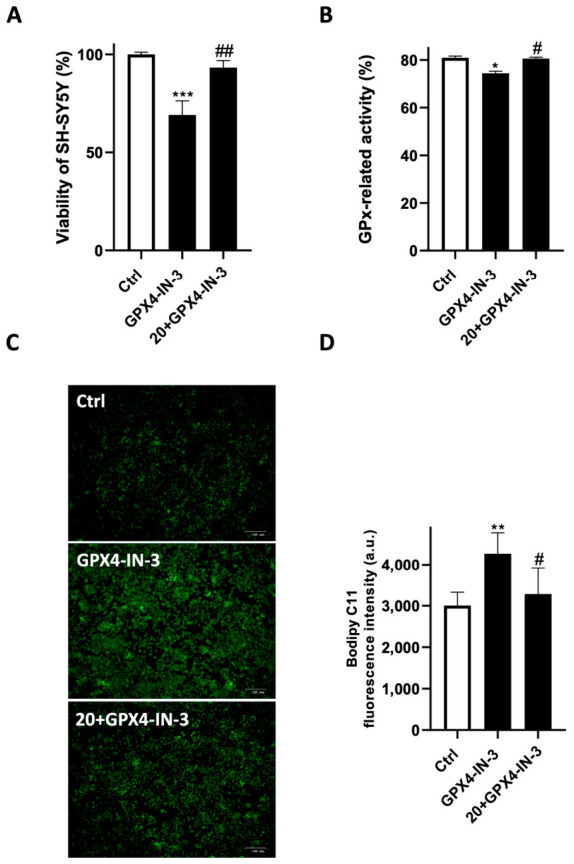
Compound 20 counteracts GPx4 inhibition-driven ferroptosis in SH-SY5Y cells. (**A**) Neuroprotective effect of compound 20 against GPX4-IN-3-treated cells evaluated by MTT assay. (**B**) Determination of GPx activity against GPX4-IN-3-treated cells evaluated via spectrophotometric assay. (**C**) Representative fluorescence microscopy images of SH-SY5Y cells stained with BODIPY C11, showing lipid peroxidation. (**D**) Quantitative analysis of oxidized BODIPY C11 fluorescence under the same experimental conditions. The changes in viability and enzymatic activity were determined by calculating the percentage of viable cells in treated cultures relative to untreated controls. Scale bar: 100 μm. *n* ≥ 5. Magnification 20×. Data are expressed as mean ± SD of three independent experiments performed in triplicate. *, **, *** denote *p* < 0.05, *p* < 0.01 and *p* < 0.001 vs. Ctrl; #, ## denote respectively *p* < 0.05 and *p* < 0.01 vs. GPX4-IN-3.

To further confirm the involvement of ferroptosis in the observed neuroprotective effects, SH-SY5Y cells were challenged with GPX4-IN-3, a pharmacological inhibitor of GPx4, the central regulator of ferroptotic cell death. Cell viability, assessed by the MTT assay, was significantly reduced upon GPx4 inhibition compared to control cells (*p* < 0.001 vs. Ctrl), consistent with induction of ferroptosis-associated cytotoxicity. Notably, co-treatment with compound 20 significantly restored cell viability relative to GPX4-inhibited cells (*p* < 0.01 vs. GPX4-IN-3), indicating a protective rescue effect (Figure 6A). The concentration of GPX4-IN-3 was preliminarily validated using the ferroptosis inhibitor ferrostatin-1 as a positive control to ensure specific ferroptosis induction.

The enzymatic assessment of GPx4 activity confirmed a significant reduction following pharmacological inhibition (*p* < 0.05 vs. Ctrl), while co-treatment with compound 20 restored functional enzymatic activity (*p* < 0.05 vs. GPX4-IN-3), supporting a protective mechanism acting on the ferroptosis pathway (Figure 6B).

To corroborate these findings at the level of lipid oxidative damage, lipid peroxidation was evaluated using the lipid-sensitive probe BODIPY C11. GPx4 inhibition induced a marked increase in oxidized lipid levels compared to control cells (a.u. Ctrl: 3002.33 ± 325.22; a.u. GPX4-IN-3: 4269.75 ± 506.06, *p* < 0.01 vs. Ctrl), whereas co-administration of compound 20 significantly reduced BODIPY C11 oxidation (a.u. 20+GPX4-IN-3: 3280.62 ± 641.99, *p* < 0.05 vs. GPX4-IN-3), restoring values close to basal conditions (Figure 6D). Representative fluorescence images were consistent with the quantitative analysis. Fluorescence images showed a pronounced accumulation of the oxidized BODIPY C11 signal in GPX4-IN-3-treated cells, consistent with ferroptosis-associated lipid peroxidation. Notably, co-treatment with compound 20 markedly reduced the intensity and distribution of the fluorescent signal, restoring a pattern similar to that observed in control cells and supporting its ability to counteract ferroptotic lipid damage (Figure 6C).

Collectively, these results demonstrate that compound 20 is able to counteract GPx4 inhibition-induced ferroptotic cell death, functionally confirming its ability to modulate ferroptosis-related pathways, in agreement with the protection observed against Aβ(25–35)-induced oxidative stress.

### 3.5. Metabolomic Investigation

Given the limited information on the metabolomic effects of ferroptotic conditions in Aβ(25–35)-exposed SH-SY5Y neuroblastoma cells, we first examined the impact of Aβ(25–35) alone on the intracellular metabolome.

**Figure 7:**
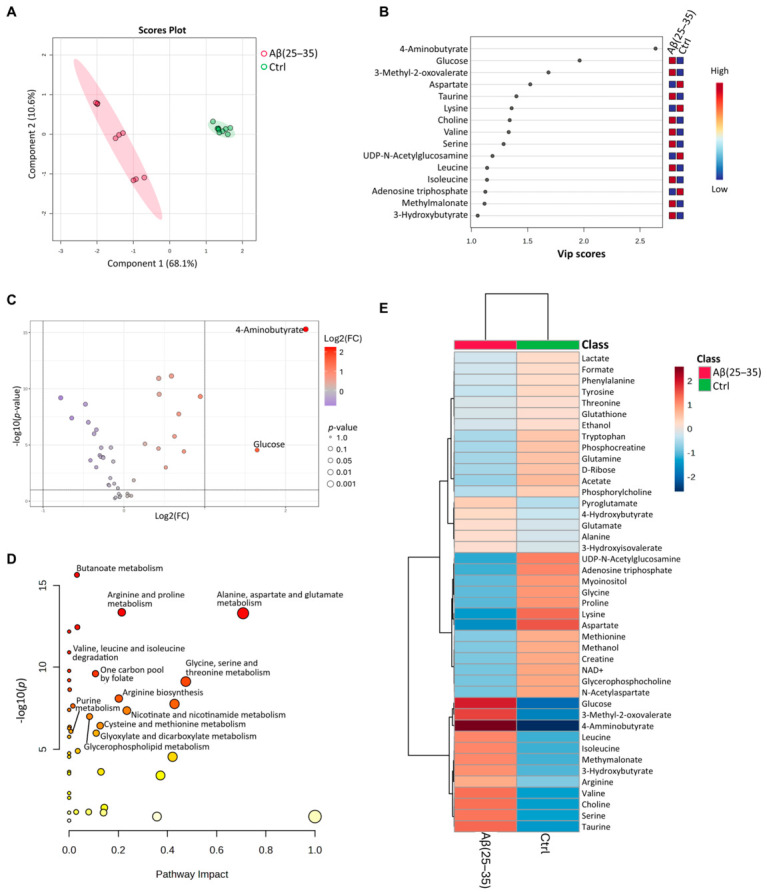
^1^H-NMR metabolomics reveals Aβ(25–35)-induced alterations in the endometabolome of SH-SY5Y cells. (**A**) PLS-DA score plot of metabolomic profiles from the endometabolome, comparing SH-SY5Y cells treated with Aβ(25–35) and control cells (Ctrl). Cluster separation is shown in Cartesian space defined by principal components PC1 (68.1%) and PC2 (10.6%). Model performance was evaluated using cross-validation (CV) based on the PLS-DA statistical protocol, revealing significant separation between groups (accuracy of 1.0 on both PC1 and PC2, Q2 of 0.93 and 0.98 on PC1 and PC2, respectively). (**B**) VIP score plot of metabolites contributing to group separation. (**C**) Volcano Plot showing metabolic changes in the cellular extract (endometabolome) of comparing SH-SY5Y cells treated with Aβ(25–35) and control cells (Ctrl). Each point represents a metabolite, plotted according to *p*-value and fold-change thresholds. The black dashed lines indicate the *p*-value and fold-change thresholds (<0.05 and ±2.0, respectively). Red points indicate upregulated metabolites; blue points indicate downregulated metabolites. (**D**) Pathway analysis showing the pathways affected by Aβ(25–35) according to *p*-values (y axis) and pathway impact values (*x*-axis). The color and size of each circle are based on *p*-values and pathway impact values, respectively. Small *p*-value and large pathway impact circles indicate that the pathway is significantly affected by the treatment. The pathways characterized by high significance and pathway impact are labeled. (**E**) Heatmap of changed metabolites in the endometabolome of SH-SY5Y cells treated with Aβ(25–35) with respect to control cells (Ctrl). The color of each section corresponds to a concentration value of each metabolite calculated by a normalized concentration matrix (red, upregulated; blue, downregulated).

We compared the metabolomic profiles of treated and control cells using PLS-DA. The score plot shown in Figure 7A clearly indicates a distinct separation between the two groups. This plot was represented in Cartesian space by the main components PC1 and PC2, which accounted for 68.1% and 10.6% of the variance, respectively. The difference between the two groups was supported by validation indices from cross-validation, with an accuracy of 1.0 for both PC1 and PC2, and Q2 values of 0.93 and 0.98 on PC1 and PC2, respectively. The metabolites responsible for this discrimination were ranked according to the VIP score, with metabolites considered significant only if VIP > 1 (Figure 7B). Upon treatment, increased intracellular levels of 4-aminobutyrate, glucose, 3-methyl-2-oxovaleric acid, taurine, choline, methylmalonate and 3-hydroxybutyrate, and decreased levels of UDP-N-acetylglutamine and adenosine triphosphate were observed. Notably, several amino acid alterations were observed with higher valine, serine, and leucine levels, along with decreased aspartate, lysine, and isoleucine levels in the endometabolome of SH-SY5Y cells exposed to Aβ(25–35) (Figure S1). The effects on 4-aminobutyrate and glucose were validated by univariate analysis with a Volcano Plot (Figure 7C).

To gain further insights, pathway analysis was performed with MetPa within MetaboAnalyst 6.0 (Figure 7D, Table S1), highlighting a significant effect on antioxidant defense pathways, including glutathione metabolism and the interconnected cysteine and methionine metabolism. Additionally, the alteration of lipid-related compounds appears as a result of the impact on glycerophospholipid metabolism, alongside pathways functionally connected to NADPH homeostasis, such as nicotinate and nicotinamide metabolism, the one-carbon pool by folate, and purine metabolism. Notably, the modulation of amino acid pathways was observed, including arginine, proline, alanine, aspartate, glutamate, glycine, serine, and threonine metabolism, together with arginine biosynthesis, as well as valine, leucine, and isoleucine degradation.

**Figure 8:**
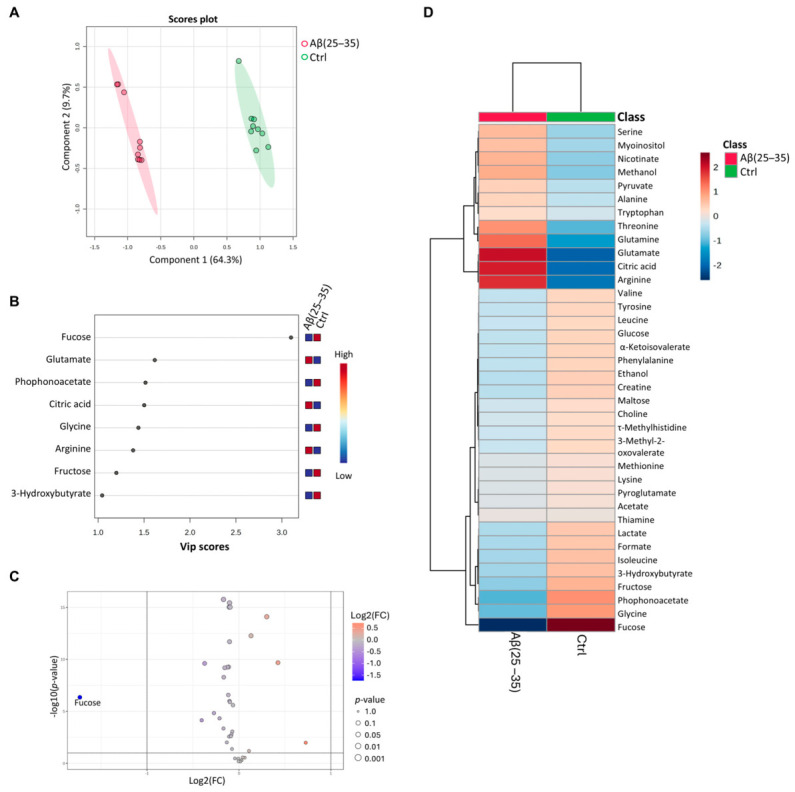
^1^H-NMR metabolomics reveals Aβ(25–35)-induced alterations in the exometabolome of SH-SY5Y cells. (**A**) PLS-DA score plot of metabolomic profiles from the exometabolome, comparing SH-SY5Y cells treated with Aβ(25–35) and control cells (Ctrl). Cluster separation is shown in Cartesian space defined by principal components PC1 (64.3%) and PC2 (9.7%). Model performance was evaluated using cross-validation (CV) based on the PLS-DA statistical protocol, revealing significant separation between groups (accuracy of 1.0 on both PC1 and PC2, Q2 of 0.96 and 0.97 on PC1 and PC2, respectively). (**B**) VIP score plot of metabolites contributing to group separation. (**C**) Volcano Plot showing metabolic changes in the growth medium (exometabolome) of comparing SH-SY5Y cells treated with Aβ(25–35) and control cells (Ctrl). Each point represents a metabolite, plotted according to *p*-value and fold-change thresholds. The black dashed lines indicate the *p*-value and fold-change thresholds (<0.05 and ±2.0, respectively). Red points indicate upregulated metabolites; blue points indicate downregulated metabolites. (**D**) Heatmap of changed metabolites in the exometabolome of SH-SY5Y cells treated with Aβ(25–35) with respect to control cells (Ctrl). The color of each section corresponds to a concentration value of each metabolite calculated by a normalized concentration matrix (red, upregulated; blue, downregulated).

The same statistical methods were used to assess whether SH-SY5Y cells exposed to Aβ(25–35) differed from untreated cells in terms of the exometabolome (Figure S1). The PLS-DA score plot in Figure 8A illustrates the separation of clusters in a Cartesian space defined by PC1 and PC2, which explain 64.3% and 9.7% of the variance, respectively. Model validation using cross-validation indicated an accuracy of 1.0 for both PC1 and PC2, along with Q2 values of 0.96 and 0.97. Figure 8B shows the VIP score plot, highlighting metabolites that distinguish the two groups. The growth medium of treated cells has higher levels of glutamate, citric acid, and arginine, and lower levels of fucose, phosphonoacetate, glycine, fructose, and 3-hydroxybutyric acid. Additionally, the Volcano Plot confirmed reduced fucose levels in cells exposed to Aβ(25–35) (Figure 8C). The color gradients in the heatmap further supported the same trend in metabolite concentration changes (Figure 8D).

**Figure 9:**
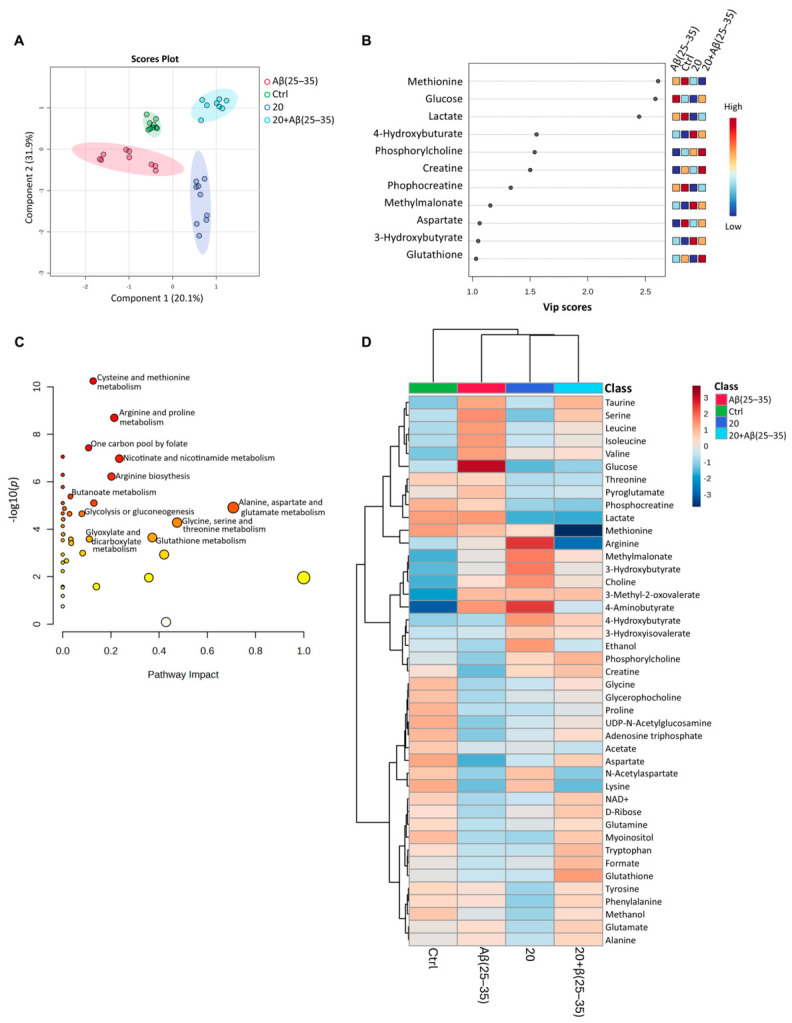
^1^H-NMR metabolomics reveals compound 20 effects in the endometabolome of SH-SY5Y cells. (**A**) PLS-DA score plot of metabolomic profiles from the endometabolome, comparing SH-SY5Y cells treated with Aβ(25–35) peptide, SH-SY5Y cells co-treated with Aβ(25–35) peptide and compound 20, SH-SY5Y cells treated with compound 20, and control cells (Ctrl). Cluster separation is shown in Cartesian space defined by principal components PC1 (20.1%) and PC2 (31.9%). Model performance was evaluated using cross-validation (CV) based on the PLS-DA statistical protocol, revealing significant separation between groups (accuracy of 0.55 on PC1 and 0.83 on PC2, Q2 of 0.79 and 0.89 on PC1 and PC2, respectively). (**B**) VIP score plot of metabolites contributing to group separation. (**C**) Pathway analysis showing the pathways affected by compound 20 in the presence of Aβ(25–35) peptide according to *p*-values (y axis) and pathway impact values (*x*-axis). The color and size of each circle are based on *p*-values and pathway impact values, respectively. Small *p*-value and large pathway impact circles indicate that the pathway is significantly affected by the treatment. The pathways characterized by high significance and pathway impact are labeled. (**D**) Heatmap of changed metabolites in the endometabolome of SH-SY5Y cells treated with Aβ(25–35) peptide, SH-SY5Y cells co-treated with Aβ(25–35) peptide and compound 20, SH-SY5Y cells treated with compound 20, and control cells (Ctrl). The color of each section corresponds to a concentration value of each metabolite calculated by a normalized concentration matrix (red, upregulated; blue, downregulated).

To analyze the impact of compound 20 on the metabolic changes caused by Aβ(25–35) ferroptosis, we conducted an ^1^H-NMR metabolomic analysis. This method has proven highly effective in revealing the mechanisms of action of different molecules, offering a comprehensive view of the biological effects triggered by a treatment. Using PLS-DA analysis, we compared the metabolic profiles of four reference groups: (i) cells treated with Aβ(25–35), (ii) cells treated with both Aβ(25–35) and compound 20, (iii) untreated cells, and (iv) cells treated only with compound 20, as reference groups. The PLS-DA results showed that the endometabolome of cells co-treated with Aβ(25–35) and compound 20 has a distinct metabolic profile from cells exposed only to Aβ(25–35), cells treated solely with compound 20, and untreated cells (Figure 9A). These differences were confirmed by cross-validation metrics, with an accuracy of 0.55 on PC1 and 0.83 on PC2, and Q2 scores of 0.79 and 0.89 on PC1 and PC2, respectively.

VIP analysis revealed that cells treated with both the Aβ(25–35) peptide and compound 20 possessed the highest levels of creatine, phosphorylcholine, and glutathione. Conversely, this group showed lower concentrations of methionine. The comparative analysis showed increased levels of 4-hydroxybutyrate, methylmalonate, aspartate, and 3-hydroxybutyrate compared to cells exposed only to Aβ(25–35), while reduced concentrations of glucose, lactate, and phosphocreatine were observed in the same group (Figure 9B).

Figure 9D displays the heatmap showing the average concentration of metabolites detected in the endometabolome of the four groups under analysis. To further explore the effects of compound 20 in the presence of Aβ(25–35), we conducted pathway analysis comparing the metabolic profiles of cells treated solely with the molecule and those treated with both Aβ(25–35) and compound 20 (Figure 9C, Table S2). The analysis highlights the modulation of pathways related to amino acids and redox homeostasis, such as arginine and proline metabolism, glycine, serine, and threonine metabolism, arginine biosynthesis, and alanine, aspartate, and glutamate metabolism. Additionally, mitochondrial energy pathways, including glycolysis and gluconeogenesis, were modulated, suggesting a restoration of the metabolic functionality of these organelles. Consistent with the increase in glutathione and the reduction in methionine, pathway analysis confirmed the effects of compound 20 on glutathione, cysteine and methionine metabolism. Furthermore, the one-carbon pool by folate, as well as nicotinate and nicotinamide metabolism, emerged as Aβ(25–35)-affected pathways that are significantly influenced by the co-presence of compound 20.

**Figure 10:**
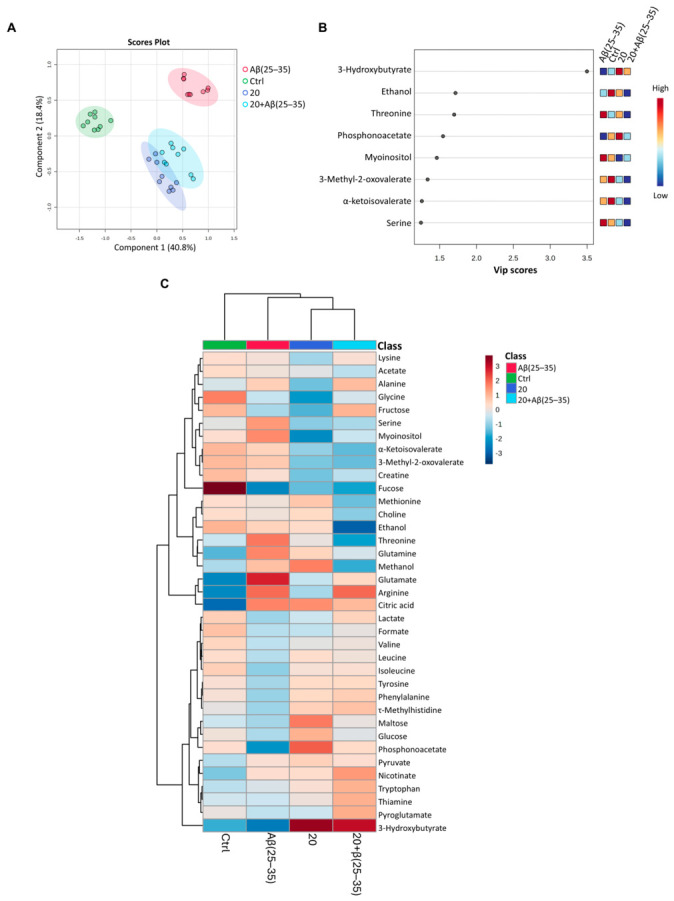
^1^H-NMR metabolomics reveals compound 20 effects in the exometabolome of SH-SY5Y cells. (**A**) PLS-DA score plot of metabolomic profiles from the exometabolome, comparing SH-SY5Y cells treated with Aβ(25–35) peptide, SH-SY5Y cells co-treated with Aβ(25–35) peptide and compound 20, SH-SY5Y cells treated with compound 20, and control cells (Ctrl). Cluster separation is shown in Cartesian space defined by principal components PC1 (20.1%) and PC2 (31.9%). Model performance was evaluated using cross-validation (CV) according to the PLS-DA statistical protocol, revealing significant separation between groups (accuracy of 0.50 on PC1 and 0.86 on PC2, Q2 values of 0.74 and 0.90 on PC1 and PC2, respectively). (**B**) VIP score plot of metabolites contributing to group separation. (**C**) Heatmap of changed metabolites in the exometabolome of SH-SY5Y cells treated with Aβ(25–35) peptide, SH-SY5Y cells co-treated with Aβ(25–35) peptide and compound 20, SH-SY5Y cells treated with compound 20, and control cells (Ctrl). The color of each section corresponds to a concentration value of each metabolite calculated by a normalized concentration matrix (red, upregulated; blue, downregulated).

To fully understand the differences among the four groups, we finally analyzed the metabolomic profile of the growth medium. The PLS-DA score plot in Figure 10A confirmed the distinction between the groups based on the exometabolome. Cross-validation results showed good accuracy and Q2 scores: 0.50 on PC1 and 0.86 on PC2 for accuracy, and 0.74 and 0.90 on PC1 and PC2 for Q2. The VIP plot highlights the metabolites most responsible for separating the clusters (Figure 10B). SH-SY5Y cells treated with both compound 20 and Aβ(25–35) have lower levels of ethanol, threonine, 3-methyl-2-oxovalerate, alpha-ketoisovalerate, and serine. The group of cells undergoing co-treatment showed a reduction in myo-inositol, while exhibiting an increase in 3-hydroxybutyrate and phosphonoacetate compared to cells exposed only to Aβ(25–35). Conversely, cells exposed solely to Aβ(25–35) peptide show very low levels of isoleucine, lactate, and leucine. The heatmap of average metabolite concentrations in the growth medium is illustrated in Figure 10C.

## 4. Discussion

Ferroptosis has increasingly emerged as a relevant contributor to neurodegenerative processes associated with amyloid toxicity, where iron dyshomeostasis, oxidative stress, and lipid peroxidation converge to promote neuronal damage [33,34,35,36]. In this regard, undifferentiated human neuroblastoma SH-SY5Y cells represent a widely used in vitro model to investigate neuronal responses to ferroptosis and oxidative stress-related mechanisms [37,38]. In particular, exposure of SH-SY5Y cells to β-amyloid peptides has been shown to induce ferroptosis-like features, including iron accumulation, glutathione depletion, and lipid peroxidation, making this system a suitable model to study ferroptosis-associated neurotoxicity and the potential neuroprotective activity of antioxidant compounds [39,40,41].

Consistent with previous studies showing that amyloid peptides can trigger lipid oxidative damage, exposure to Aβ(25–35) significantly increased MDA levels and oxidized BODIPY C11 fluorescence in SH-SY5Y cells. These effects were also confirmed by the metabolomics results: the treatment of SH-SY5Y cells with Aβ(25–35) influences glycerophospholipid metabolism, which is among the most affected biochemical pathways. Glycerophospholipids constitute approximately half of the lipids in the brain, playing a crucial role in membrane stability. Among the lipid classes, polyunsaturated glycerophospholipids (PUFA-GPLs), specifically those containing phosphatidylethanolamine (PUFA-PEs), have been identified as the selective targets of pro-ferroptotic lipid peroxidation [42,43]. The changes observed in glycerophospholipid metabolism align with the metabolic reprogramming linked to ferroptotic stress. This pattern may indicate a compensatory activation of lipid-remodeling pathways, attempting to replenish phospholipids that are increasingly depleted by iron-induced lipid peroxidation. Altogether, these alterations confirm the strong pro-oxidant effect of amyloid fragments and their ability to induce ferroptosis in neuronal models. Importantly, co-treatment with compound 20 markedly reduced both TBARS-derived MDA formation and lipid ROS accumulation, indicating efficient suppression of lipid peroxidation. Interestingly, MDA levels in co-treated cells were slightly lower than those observed in control cells. This “extra-protective” effect suggests that compound 20 does not merely neutralize Aβ-induced damage but may also enhance the basal antioxidant capacity of SH-SY5Y cells. This is likely achieved through its multifaceted action, which includes the reduction in the labile iron pool and the robust restoration of intracellular glutathione levels, potentially mitigating even physiological levels of lipid peroxidation. These findings, in accordance with our previous work, corroborate the antioxidant properties of compound 20 [4], which can intercept oxidative cascades upstream of membrane lipid damage, thereby limiting one of the central drivers of ferroptotic cell death.

In parallel, the restoration of intracellular glutathione levels and GPx4 activity represent crucial determinants of ferroptosis resistance. The glutathione system plays a pivotal role in detoxifying lipid peroxides via glutathione peroxidases, particularly GPx4, and its depletion is widely recognized as a key trigger of ferroptotic vulnerability [44,45,46]. In the present study, Aβ(25–35) exposure resulted in a significant reduction in intracellular GSH levels and GPx activity, confirming the impairment of antioxidant defenses in amyloid-stressed cells. Further confirmation of this system’s disruption in the presence of Aβ(25–35) is the extracellular increase in glutamate, which has been linked to the inhibition of cystine uptake via system xCT, leading to reduced GSH levels and promoting ferroptosis [47]. High levels of Aβ peptides are known to impair neuronal glutamate uptake, causing glutamate to build up in the synaptic space [14,48,49,50]. This can indirectly affect ferroptosis-related pathways by restricting cystine intake through system xCT. When cystine is limited, intracellular glutathione levels decrease, affecting glutathione metabolism and promoting broader metabolic changes that increase susceptibility to ferroptosis. Remarkably, compound 20 efficiently restored GSH levels, as demonstrated by the recovery of mBCl fluorescence intensity and confirmed by direct GSH concentration measurement obtained in the metabolomic analysis. The rise in GSH levels indicates that compound 20 can effectively restore intracellular GSH reserves, offering protection against metabolic changes associated with ferroptosis.

The modulation of glutathione metabolism is reflected in altered levels of methionine and corresponding changes in the methionine and cysteine pathways. Methionine is the precursor of S-adenosylmethionine (SAM), which can be converted into cysteine through the folate-dependent transsulfuration pathway [51,52], which is modulated by compound 20. We can therefore speculate that the reduced methionine levels reflect an increased flux toward cysteine biosynthesis to sustain higher glutathione production. Aβ(25–35) disrupts the folate cycle, thereby interfering with the methionine–homocysteine cycle and elevating cellular NADPH demand, ultimately decreasing NADPH availability. Since NADPH is vital for maintaining glutathione in its reduced form and supporting GPx4 activity, its depletion heightens vulnerability to ferroptosis, characterized by iron-dependent lipid peroxidation [53,54]. Conversely, compound 20 restores the folate cycle and enhances glutathione availability. Thus, reduced methionine levels likely indicate an increased flux toward cysteine biosynthesis to support higher glutathione production. The action of compound 20 on GSH homeostasis was further supported by gene expression analysis, which showed the recovery of GPx4 expression in the co-treatment condition. Together, these observations indicate that compound 20 not only scavenges reactive species but also preserves glutathione-dependent redox buffering systems and enhances GSH production, thereby reinforcing cellular antioxidant capacity.

Another critical feature of ferroptosis is iron dysregulation, which promotes the formation of highly reactive oxygen species through the Fenton reaction and amplifies lipid peroxidation. In agreement with this mechanism, Aβ(25–35) treatment significantly increased intracellular labile Fe^2+^ levels, as revealed by FerroOrange staining. This finding supports the notion that amyloid toxicity is associated with alterations in neuronal iron homeostasis. Notably, compound 20 significantly reduced intracellular ferrous iron accumulation, restoring fluorescence signals to control levels. The reduction in the labile iron pool may limit oxidative propagation reactions and thus attenuate ferroptotic signaling [55,56,57].

Oxidative stress and iron dysregulation are tightly linked to mitochondrial dysfunction and endoplasmic reticulum (ER) stress, collectively contributing to ferroptosis-associated neurodegeneration [58,59,60,61,62,63]. Mitochondria are highly sensitive to redox imbalance and lipid peroxidation, and their functional impairment can further exacerbate oxidative stress and metabolic dysregulation. In the present study, Aβ(25–35) induced alterations in mitochondrial membrane potential, reflected by increased and diffuse TMRE fluorescence. This abnormal signal likely reflects dysfunctional mitochondrial polarization and impaired bioenergetic regulation, rather than enhanced mitochondrial activity. The disruption of normal mitochondrial bioenergetic function is corroborated by decreased adenosine triphosphate (ATP) levels in the endometabolome of cells exposed to Aβ(25–35), consistent with recent multiomics studies [5]. Importantly, compound 20 was able to normalize TMRE fluorescence patterns, suggesting preservation of mitochondrial integrity under amyloid-induced stress conditions. Metabolomic data confirm the modulation of mitochondrial activity, showing that compound 20, in the presence of Aβ(25–35), influences glycolysis and gluconeogenesis, ultimately improving bioenergetics. These impacts on cellular metabolism suggest that compound 20 can trigger metabolic reprogramming to reestablish the energy metabolism of SH-SY5Y cells that have been compromised by mitochondrial damage associated with ferroptosis.

Additionally, several mitochondrial-linked amino acid pathways such as alanine, aspartate, and glutamate metabolism, as well as proline and arginine metabolism, are affected. The capacity of compound 20 to modulate proline and arginine metabolism, processes often disrupted in neurodegenerative diseases, is a trait common to other natural molecules that exhibit protective effects in in vitro systems against Aβ(25–35) [11,64,65]. This indicates potential broader impacts on redox balance and reactive oxygen species (ROS) production.

The functional interplay between mitochondria and the endoplasmic reticulum represents another key aspect of cellular stress responses. ER stress and activation of the unfolded protein response (UPR) have been widely implicated in neurodegenerative disorders and are closely linked to oxidative stress and calcium dysregulation. Consistent with this scenario, Aβ(25–35) treatment markedly increased ER-ID fluorescence intensity and induced transcriptional activation of UPR-related genes, including IRE1, PERK, and CHOP. These molecular alterations indicate activation of ER stress signaling pathways. Co-treatment with compound 20 significantly attenuated ER expansion and reduced the expression of these stress markers, suggesting that the compound helps maintain ER homeostasis during amyloid treatment.

The molecular analysis performed in this study further supports the broad regulatory effects of compound 20 on cellular stress pathways. Aβ(25–35) exposure triggered robust activation of NRF2-driven antioxidant and inflammatory signaling pathways, including NF-κB and iNOS. While the activation of NRF2-related genes such as HO-1, NQO1 and SOD1 may initially represent an adaptive response to oxidative stress, their sustained upregulation reflects persistent cellular damage. Notably, compound 20 markedly attenuated the overexpression of these stress-responsive genes, suggesting that it mitigates upstream triggers of oxidative and inflammatory signaling rather than simply enhancing downstream antioxidant responses. This interpretation is consistent with the observed reduction in lipid peroxidation, restoration of glutathione levels, and normalization of iron homeostasis.

The metabolic profile supports the restoration of both redox and inflammatory balance. Aβ(25–35) induces a glycolytic shift, characterized by reduced intracellular glucose and increased lactate as part of an NRF2-dependent redox adaptation. In contrast, compound 20 counteracts this metabolic reprogramming, as demonstrated by higher intracellular glucose levels, lower lactate levels, and a marked reduction in NRF2 expression. The change in the glucose-to-lactate ratio indicates that glycolytic activity returns to normal levels, and the cells rely less on glycolysis to sustain redox balance. Pathway analysis further supports this interpretation, showing that compound 20 promotes a metabolic reprogramming that reduces redox imbalance and attenuates inflammatory signaling, both of which are known to exacerbate ferroptotic susceptibility [66,67].

To further strengthen the mechanistic interpretation of these findings, rescue experiments under GPx4 inhibition were performed, confirming that compound 20 retains its protective effects under ferroptosis-sensitizing conditions. Collectively, these data support the conclusion that its activity is functionally linked to the modulation of ferroptosis susceptibility rather than a non-specific antioxidant response.

## 5. Conclusions

In this study, we provide a comprehensive and integrated characterization of the neuroprotective activity of compound 20 against Aβ (25–35) induced cellular stress in SH-SY5Y cells, expanding and mechanistically reinforcing our previous findings on its antioxidant and anti-amyloid properties. While our earlier work demonstrated that compound 20 modulates ROS levels, interferes with Aβ aggregation, and preserves neuronal viability, the present study reveals that its protective efficacy extends well beyond conventional disaggregating activity. Here, we showed that Aβ(25–35) triggers a complex, self-amplifying stress network involving redox imbalance, glutathione depletion, intracellular iron accumulation, lipid peroxidation, mitochondrial dysfunction, and endoplasmic reticulum stress, which collectively converge into a ferroptosis cascade. By combining biochemical assays, organelle-specific analyses, and gene expression profiling with metabolomics, we demonstrate that compound 20 effectively counteracts each of these interconnected stress nodes. Notably, compound 20 restores intracellular glutathione levels, attenuates iron dyshomeostasis, limits iron-driven lipid peroxidation, and preserves both mitochondrial membrane potential and ER homeostasis. At the transcriptional level, compound 20 markedly suppresses the Aβ-induced activation of antioxidant stress responses, inflammatory signaling, and unfolded protein response pathways, while restoring glutathione-dependent defenses, further supporting a normalization of cellular homeostasis rather than a mere stress compensation.

Importantly, the multimodal neuroprotective profile of compound 20 is further corroborated by untargeted metabolomic analysis, which reveals a profound restoration of the cellular metabolic fingerprint. Exposure to Aβ(25–35) induces a significant metabolic shift, characterized by perturbations in cysteine and methionine metabolism and in arginine and proline metabolism, in line with observed glutathione depletion and redox imbalance. Co-treatment with compound 20 effectively reverts these metabolic hallmarks, promoting a shift in the global metabolomic profile back toward the control state. Specifically, compound 20 normalizes the levels of key metabolites involved in antioxidant defense and energy production, such as methionine and glucose. This metabolic recovery perfectly aligns with the biochemical evidence of reduced lipid peroxidation, restored mitochondrial membrane potential, and attenuated ER stress. Collectively, these integrated findings demonstrate that compound 20 does not act merely as a scavenger, but as a comprehensive metabolic modulator capable of interrupting Aβ-induced ferroptosis and re-establishing cellular homeostatic integrity. These findings strengthen the rationale for further preclinical development of compound 20 as a multitarget agent for the modulation of amyloid-driven neurodegenerative processes.

The use of a single effective concentration (30 μM), selected on the basis of our previous work, where related compounds showed activity only within this range, is among the main limitations of the study. However, lower concentrations demonstrated to be ineffective and higher doses were considered potentially associated with non-specific effects. Nevertheless, this approach allowed us to focus on the mechanistic characterization of compound 20, providing a basis for future structure–activity relationship studies and the rational design of more potent analogs, which will be subjected to full dose–response characterization once their primary molecular targets have been more clearly defined.

## Figures and Tables

**Table 1:** Forward and reverse primer sequences for target genes in real-time PCR.

Primer Sequence (5′–3′)		
Target Gene	Forward	Reverse
*NRF2*	ACACGGTCCACAGCTCATC	TGTCAATCAAATCCATGTCCTG
*HO-1*	CTCAACATCCAGCTCTTTGAG	AATCTTGCACTTTGTTGCTGGC
*NQO1*	GACATCACAGGTAAACTGAAGG	GCAGGGGGAACTGGAATATC
*NF-κB*	CCCCACGAGCTTGTAGGAAAG	CCAGGTTCTGGAAACTGTGGAT
*iNOS*	ATGTCCGAAGCA AACATCAC	TAATGTCCAGGAAGTAGGTG
*GPx4*	CCTCAAGTACGTCCGACCTG	CAATGTCGTTGCGGCACACC
*SOD1*	AGGGAACCATCCACTTCGAG	TGCGCAATCCCAATCACTCC
*BIP*	CGGGCAAAGATGTCAGGAAAG	TTCTGGACGGGCTTCATAGTAGAC
*IRE1α*	CTCTGTCCGTACCGCCC	GAAGCGTCACTGTGCTGGT
*ATF6*	TTGACATTTTTGGTCTTGTGG	GCAGAAGGGGAGACACATTT
*CHOP*	ACCAAGGGAGAACCAGGAAACG	TCACCATTCGGTCAATCAGAGC

## Data Availability

The original contributions presented in this study are included in the article/Supplementary Materials. Further inquiries can be directed to the corresponding authors.

## Supplementary Materials

The following supporting information can be downloaded at https://www.mdpi.com/article/10.3390/antiox15070798/s1: Figure S1: **A** 1D ^1^H NOESY spectrum showing the polar cellular extracts (endometabolome) derived from SH-SY5Y cells. A total of 43 metabolites were identified in the endometabolome. **B** 1D ^1^H NOESY spectrum of growth medium (exometabolome) derived from SH-SY5Y cells; Table S1: Pathway analysis of the biochemical pathways affected by Aβ(25–35); Table S2: Pathway analysis of the biochemical pathways affected by compound 20 in presence of Aβ(25–35); Figure S2: Representative images of SH-SY5Y cells showing intracellular reduced glutathione (GSH) levels detected with monochlorobimane (mBCl); Figure S3: Representative fluorescence microscopy images of SH-SY5Y cells stained with FerroOrange for labile ferrous iron (Fe^2+^) detection; Figure S4. Representative fluorescence microscopy images of SH-SY5Y cells stained with BODIPY C11, showing lipid peroxidation after 24h of treatment; Figure S5: Representative fluorescence microscopy images of TMRE-stained cells to assess mitochondrial membrane potential; Figure S6: Representative fluorescence microscopy images of ER-ID-stained cells to evaluate endoplasmic reticulum (ER) expansion after 24h of treatment; Figure S7: Representative fluorescence microscopy images of SH-SY5Y cells stained with BODIPY C11, showing lipid peroxidation after 16h of treatment after GPX4-IN-3 treatment.
