# Supplementary material for: Metabolic Reprogramming Associated with Ferroptosis Protection by an Indole-Based Antioxidant in Aβ(25–35)-Treated SH-SY5Y Cells
Source: Antioxidants (Basel). 2026 Jun 26;15(7):798. doi: 10.3390/antiox15070798 (PMC13404325; doi:10.3390/antiox15070798)
Supplement: Supplementary file 1 [file antioxidants-15-00798-s001.zip › antioxidants-4293500-supplementary.pdf]

Supplementary Materials

Metabolic reprogramming associated with ferroptosis protection by an indole-based antioxidant in Aβ(25–35)-treated SH-SY5Y cells

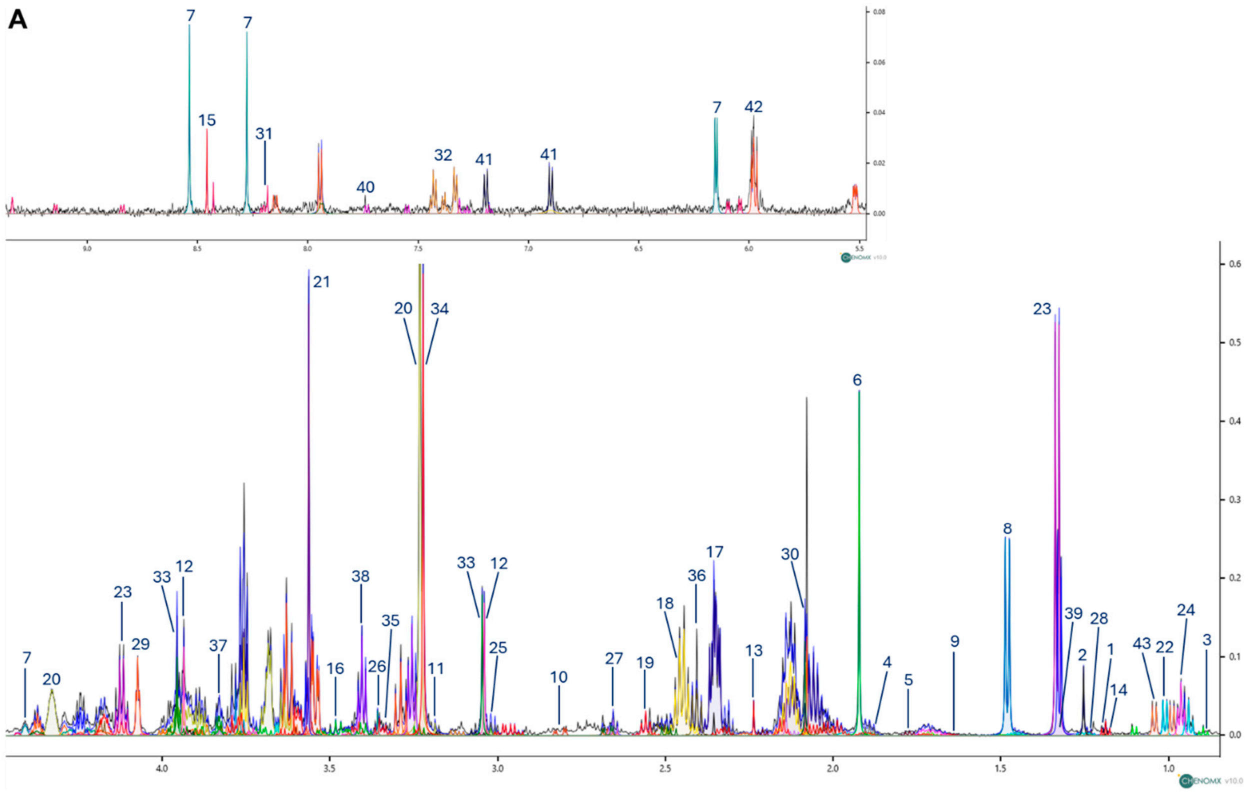

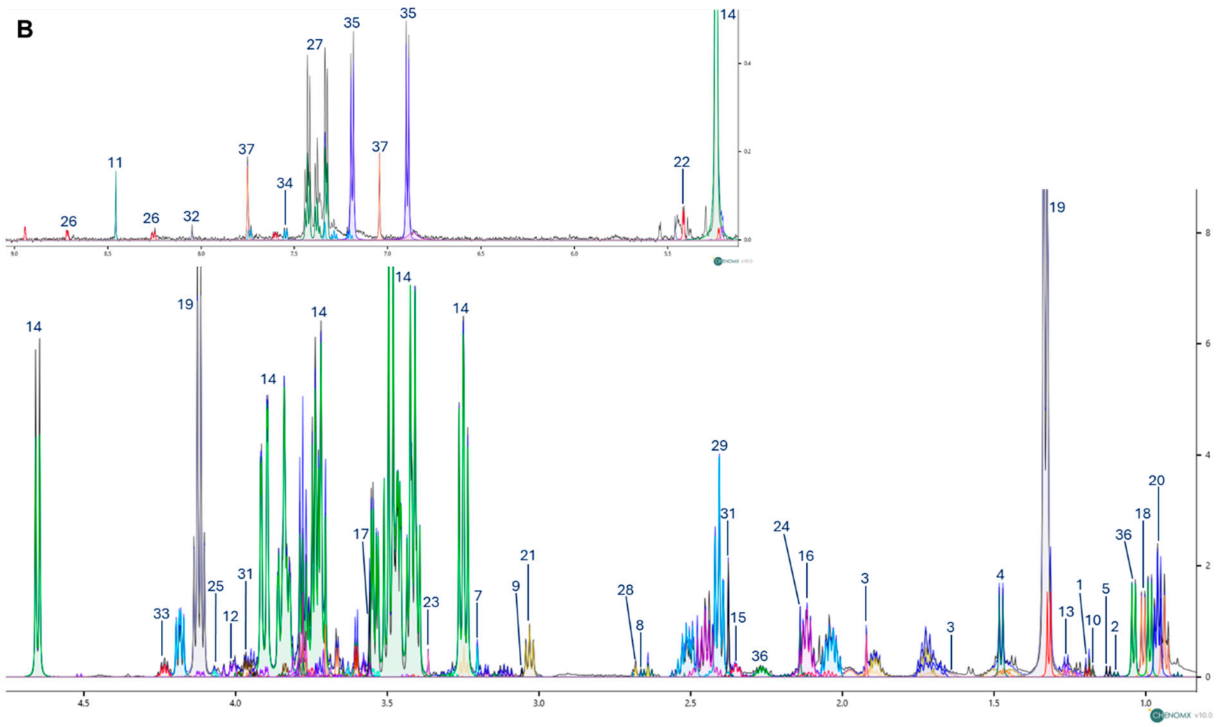

Figure S1. A 1D ^{1}H NOESY spectrum showing the polar cellular extracts (endometabolome) derived from SH-SY5Y cells. A total of 43 metabolites were identified in the endometabolome. The numbers indicate representative resonances of the following metabolites: 1: 3-hydroxybutyrate; 2: 3-Hydroxyisovaleric acid; 3: 3-Methyl-2-oxovaleric acid; 4: 4-Aminobutyrate; 5: 4-Hydroxybutyrate; 6: Acetate; 7: Adenosine triphosphate; 8: Alanine; 9: Arginine; 10: Aspartate; 11: Choline; 12: Creatine; 13: D-Ribose; 14: Ethanol; 15: Formate; 16: Glucose; 17: Glutamate; 18: Glutamine; 19: Glutathione; 20: Glycerophosphocholine; 21: Glycine; 22: Isoleucine; 23: Lactate; 24: Leucine; 25: Lysine; 26: Methanol; 27: Methionine; 28: Methylmalonate; 29: Myoinositol; 30: N-Acetylaspartate; 31: NAD+; 32: Phenylalanine; 33: Phosphocreatine; 34: Phosphorylcholine; 35: Proline; 36: Pyroglutamate; 37: Serine; 38: Taurine; 39: Threonine; 40: Tryptophan; 41: Tyrosine; 42: UDP-N-Acetylglucosamine; 43: Valine. **B** 1D ^{1}H NOESY spectrum of growth medium (exometabolome) derived from SH-SY5Y cells. The numbers indicate representative resonances of the following metabolites:1: 3-Hydroxybutyrate; 2: 3-Methyl-2-oxovalerate; 3: Acatate; 4: Alanine; 5: Alpha-ketoisovaleric acid; 6: Arginine; 7: Choline; 8: Citric acid; 9: Creatine; 10: Ethanol; 11: Formate; 12: Fructose; 13: Fucose; 14: Glucose; 15: Glutamate; 16: Glutamine; 17: Glycine; 18: Isoleucine; 19: Lactate; 20: Leucine; 21: Lysine; 22: Maltose; 23: Methanol; 24: Methionine; 25: Myoinositol; 26: Nicotinate; 27: Phenylalanine; 28: Phosphonoacetate; 29: Pyroglutamate; 30: Pyruvate; 31: Serine; 32: Thiamine; 33: Threonine; 34: Tryptophan; 35: Tyrosine; 36: Valine; 37: τ-Methylhistidine.

Table S1. Pathway analysis of the biochemical pathways affected by Aβ(25-35). The number of hits corresponds to the number of metabolites detected in the spectrum that participate in the biochemical pathways and are explicit in the column 'metabolites'. Raw p represents the significance validation index reporting the p-value; Holm p represents the adjustment of the p-value for the number of analysed samples, using Holm Bonferroni correction; the FDR index calculates the number of False Discovery Rates. Biochemical pathways with hits>2 and Raw.p, Holm p, FDR <0.05 were considered significant.

Biochemical pathway	***Hits***	***Raw p***	***Holm p***	***FDR***	***Impact***	***Metabolites***
Butanoate metabolism	3	2,25E-16	9,68E-15	9,68E-15	0,03175	3-Hydroxybutanoate;4-Amminobutanoate; Glutamate

Arginine and proline metabolism	6	4,73E-14	1,99E-12	7,74E-13	0,21395	L-Arginine;Glutamate;Creatine; 4-Amminobutanoate; Proline;Phosphocreatine
Alanine, aspartate and glutamate metabolism	6	5,40E-14	2,21E-12	7,74E-13	0,70754	NAA;Aspartate;Alanine; Glutamate;4-Amminobutanoate; Glutamine
Valine, leucine and isoleucine degradation	5	3,79E-13	1,52E-11	4,07E-12	0,03348	(S)-3-Methyl-2-oxopentanoic acid;L-Valine; L-Isoleucine;L-Leucine; Methylmalonate
One carbon pool by folate	5	2,53E-10	9,11E-09	1,36E-09	0,10714	Glycine; ATP; L-Methionine;Choline; L-Serine
Glycine, serine and threonine metabolism	5	7,66E-10	2,60E-08	3,29E-09	0,4744	L-Serine; Choline;Glycine;Threonine;Creatine
Arginine biosynthesis	4	8,23E-09	2,63E-07	2,95E-08	0,20213	L-Glutamate; L-Arginine;L-Aspartate;L-Glutamine
Nicotinate and nicotinamide metabolism	2	4,43E-08	1,20E-06	1,12E-07	0,23465	L-Aspartate;NAD
Glycerophospholipid metabolism	3	1,02E-07	2,64E-06	2,43E-07	0,08288	Choline phosphate; Choline; sn-Glycero-3-phosphocholine
Cysteine and methionine metabolism	2	3,67E-07	9,18E-06	8,31E-07	0,1263	L-Serine; L-Methionine
Purine metabolism	2	8,14E-07	1,79E-05	1,59E-06	0,0071	L-Glutamine;ATP
Glyoxylate and dicarboxylate metabolism	6	1,05E-06	2,19E-05	1,95E-06	0,11	L-Serine;Glycine;L-Glutamate;Acetate;Formate;L-Glutamine

Table S2. Pathway analysis of the biochemical pathways affected by compound 20 in the presence of Aβ(25-35). The number of hits corresponds to the number of metabolites detected in the spectrum that participate in the biochemical pathways and are explicit in the column 'metabolites'. Raw p represents the significance validation index reporting the p-value; Holm p represents the adjustment of the p-value for the number of analysed samples, using Holm Bonferroni correction; the FDR index calculates the number of False Discovery Rates. Biochemical pathways with hits>2 and Raw.p, Holm p, FDR <0.05 were considered significant.

Biochemical pathway	***Hits***	***Raw p***	***Holm p***	***FDR***	***Impact***	***Metabolites***
Cysteine and methionine metabolism	2	5,73E-11	2,46E-09	2,46E-09	0,1263	L-Serine; L-Methionine
Arginine and proline metabolism	6	2,02E-09	8,48E-08	4,34E-08	0,21395	L-Arginine;Glutamate;Creatine; 4-Amminobutanoate; Proline;Phosphocreatine
One carbon pool by folate	5	3,74E-08	1,53E-06	5,37E-07	0,10714	Glycine; ATP; L-Methionine;Choline; L-Serine
Nicotinate and nicotinamide metabolism	2	1,07E-07	4,16E-06	9,18E-07	0,23465	L-Aspartate;NAD
Arginine biosynthesis	4	6,05E-07	2,24E-05	3,72E-06	0,20213	L-Glutamate; L-Arginine;L-Aspartate;L-Glutamine

Butanoate metabolism	3	4,20E-06	0,000147	2,01E-05	0,03175	3-Hydroxybutanoate;4-Amminobutanoate; Glutamate
Alanine, aspartate and glutamate metabolism	6	1,23E-05	0,000393	4,40E-05	0,70754	NAA; Aspartate; Alanine; Glutamate;4-Amminobutanoate; Glutamine
Glycolysis or Gluconeogenesis	3	2,24E-05	0,000649	6,01E-05	0,02831	Ethanol; Lactate; Acetate
Pyruvate metabolism	3	2,24E-05	0,000649	6,01E-05	0,07951	Lactate; Acetate;Ethanol
Glycine, serine and threonine metabolism	5	5,25E-05	0,001364	0,000125	0,4744	L-Serine; Choline;Glycine;Threonine;Creatine
Glutathione metabolism	4	0,000224	0,004927	0,000438	0,37144	Gluthathione; Glycine; Glutamate; 5-Oxoproline
Glyoxylate and dicarboxylate metabolism	6	0,000259	0,005431	0,000479	0,11	L-Serine;Glycine;L-Glutamate;Acetate;Formate;L-Glutamine

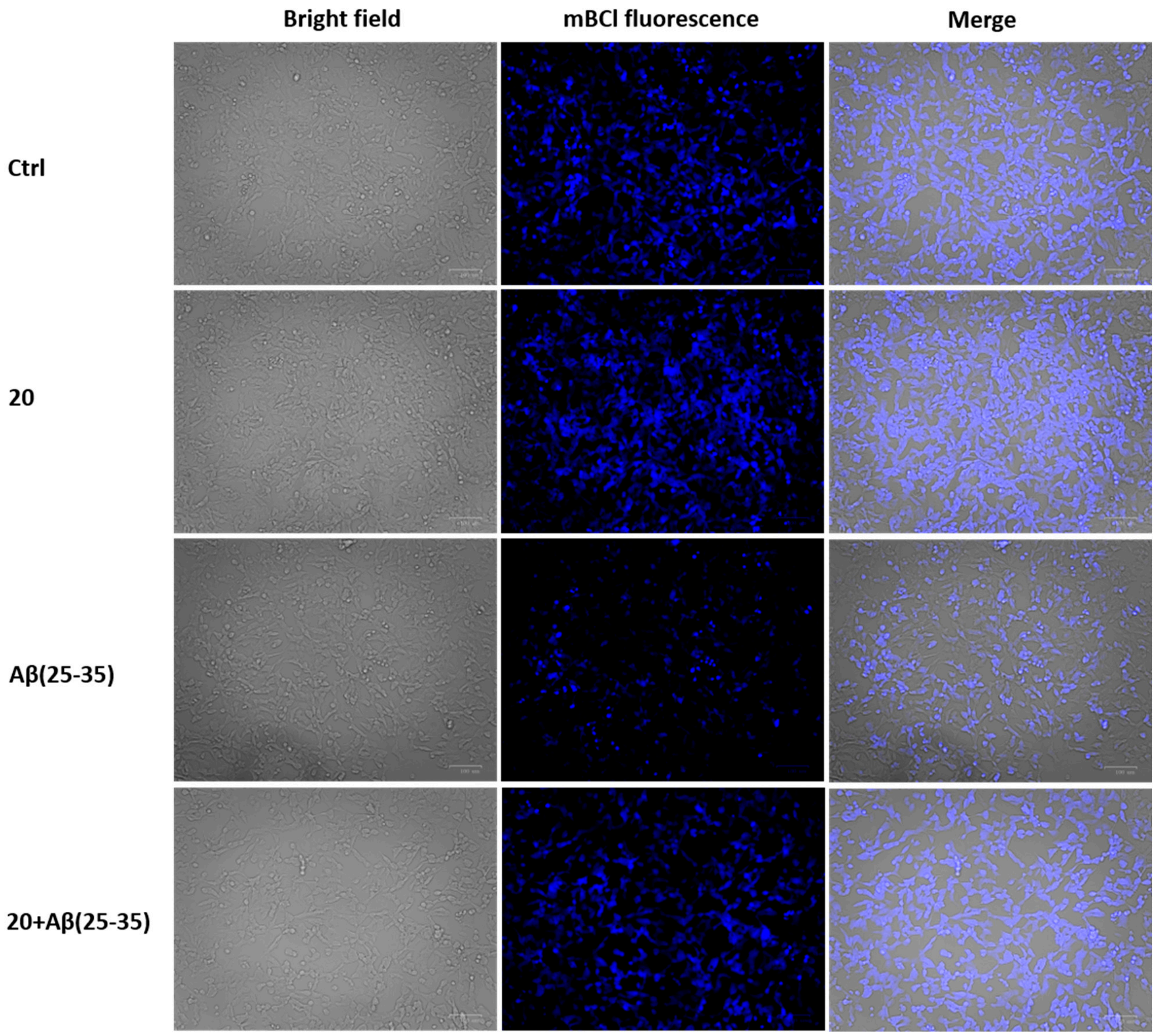

Figure S2. Representative images of SH-SY5Y cells showing intracellular reduced glutathione (GSH) levels detected with monochlorobimane (mBCl). Cells were incubated with mBCl (blue) to detect the GSH-monochlorobimane adduct after 24 h of treatment. To ensure comparable cell density across groups, each panel presents brightfield (cell morphology), mBCl fluorescence (blue), and merge images. From top to bottom: untreated control cells (Ctrl); compound 20 (30 μM); Aβ(25-35) (40 μM); and co-treatment (20 + Aβ(25-35)). Scale bar: 100 μm. N ≥ 5. Magnification 20×.

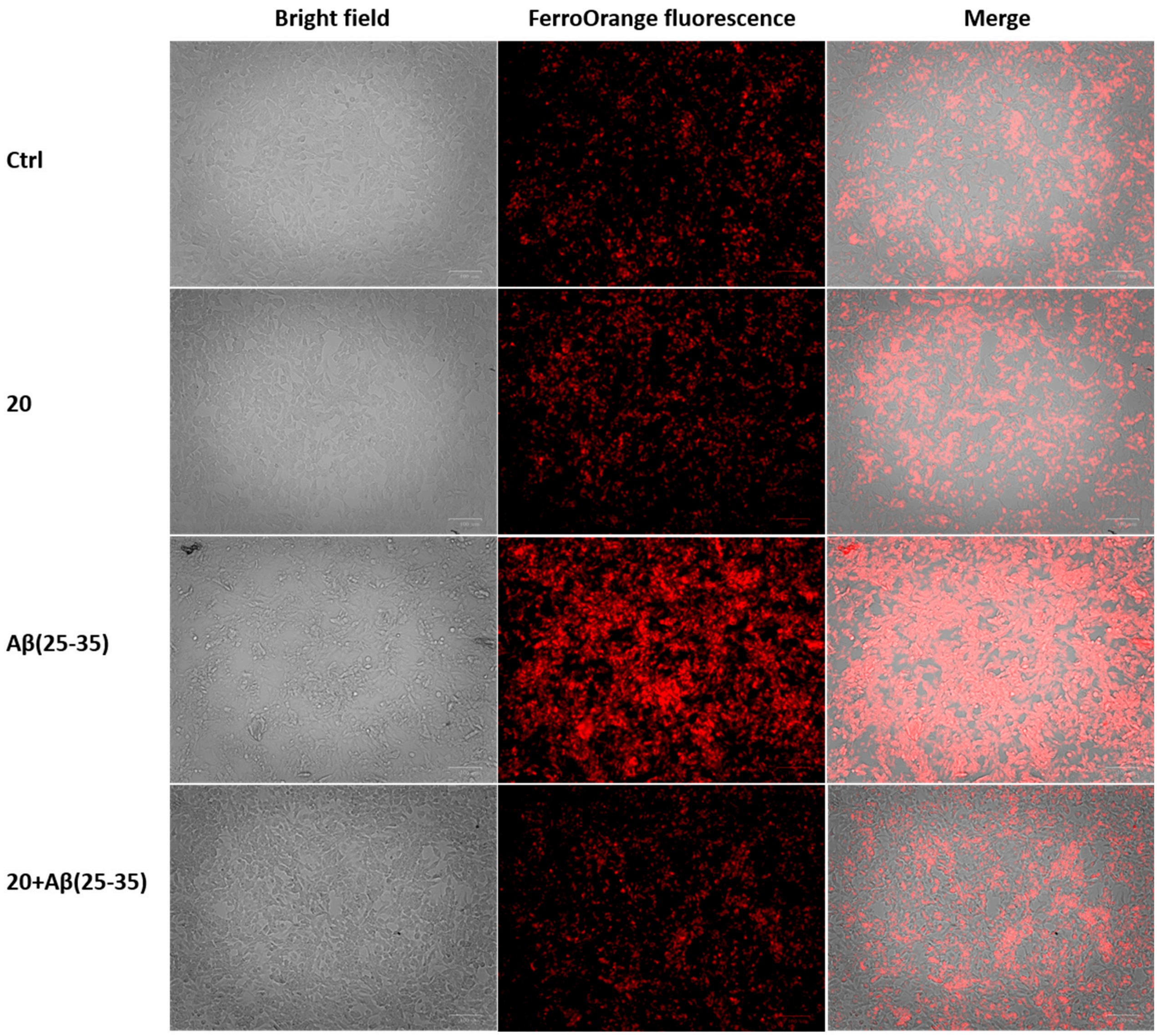

Figure S3. Representative fluorescence microscopy images of SH-SY5Y cells stained with FerroOrange for labile ferrous iron (Fe^{2+}) detection. The images were acquired after 24 h of treatment. The panel includes brightfield (morphology), FerroOrange fluorescence (red), and merge (red+brightfield). From top to bottom: untreated control cells (Ctrl); compound 20 (30 μM); Aβ(25-35) (40 μM); and co-treatment (20 + Aβ(25-35)). Scale bar: 100 μm. N ≥ 5. Magnification 20×.

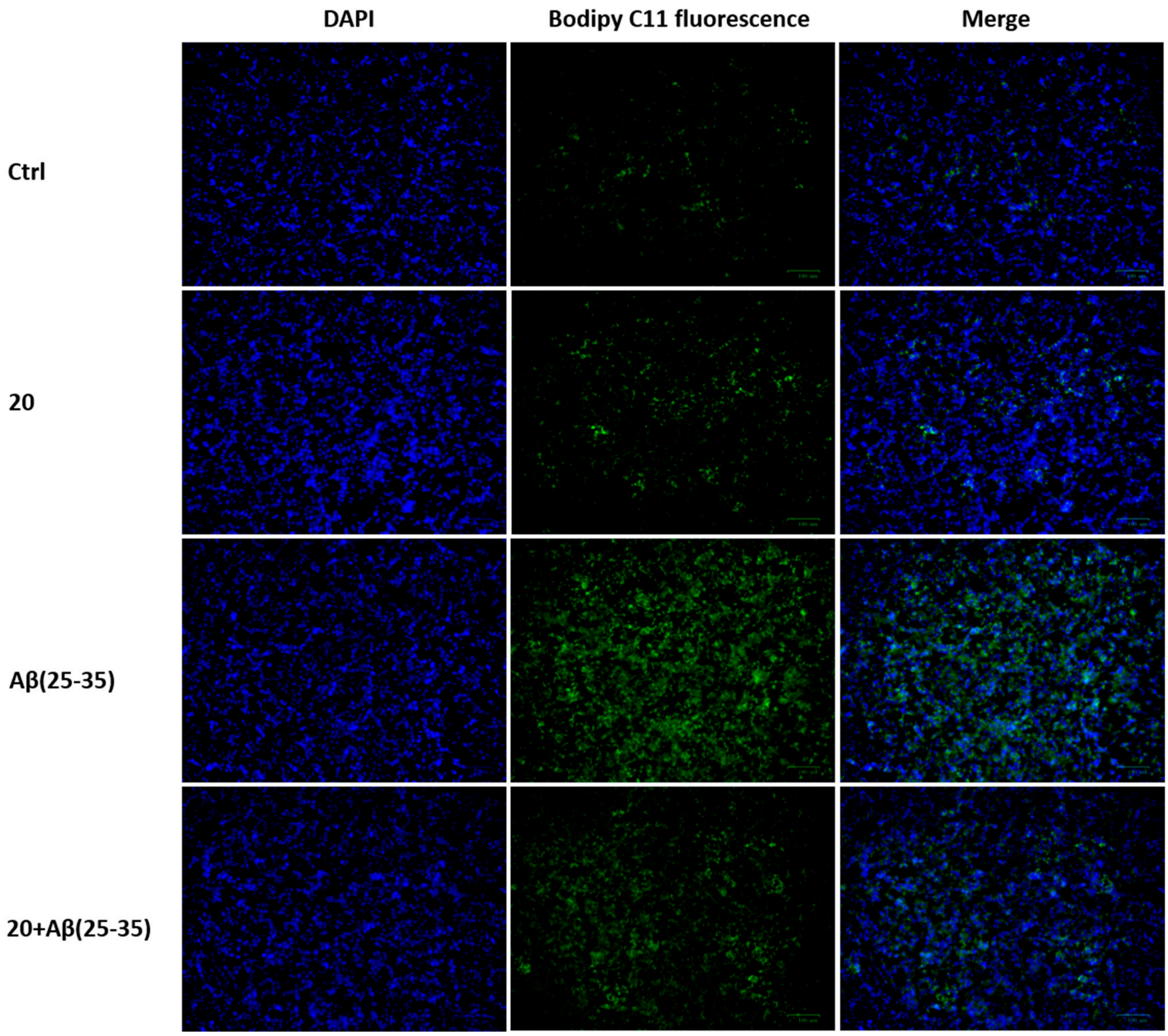

Figure S4. Representative fluorescence microscopy images of SH-SY5Y cells stained with BODIPY C11, showing lipid peroxidation after 24h of treatment. Nuclei were simultaneously stained with DAPI (blue). The panels include DAPI (nuclei, blue), oxidised BODIPY C11 (lipid ROS, green), and merge (DAPI + green). From top to bottom: untreated control cells (Ctrl); compound 20 (30 μM); Aβ(25-35) (40 μM); and co-treatment (20 + Aβ(25-35)). Scale bar: 100 μm. N ≥ 5. Magnification 20×.

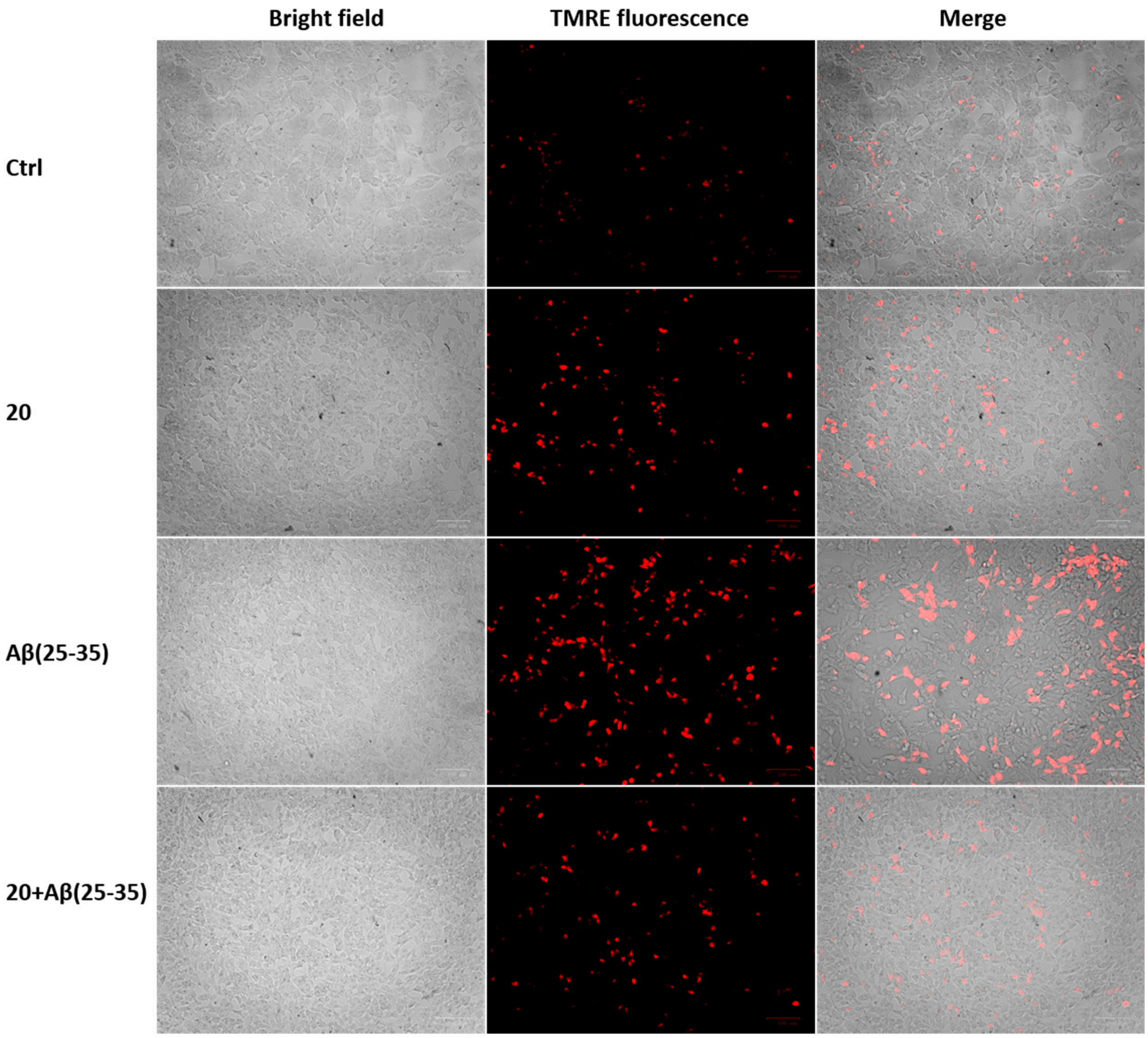

Figure S5. Representative fluorescence microscopy images of TMRE-stained cells to assess mitochondrial membrane potential. Experimental conditions were maintained for 24 h before imaging. Panels include brightfield, TMRE fluorescence (red), and merge (red + brightfield). From top to bottom: untreated control cells (Ctrl); compound 20 (30 μM); Aβ(25-35) (40 μM); and co-treatment (20 + Aβ(25-35)). Scale bar: 100 μm. N ≥ 5. Magnification 20×.

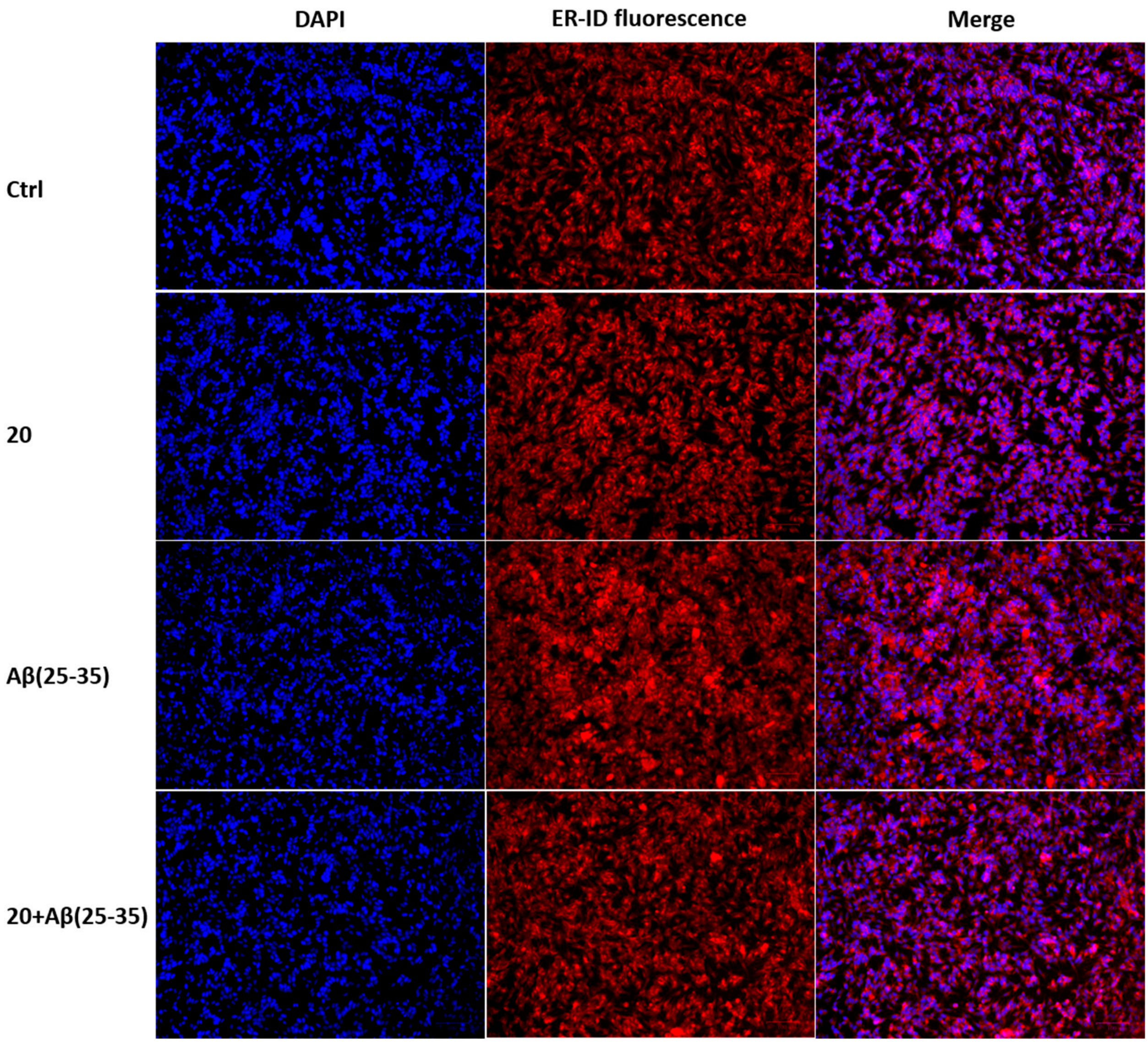

Figure S6. Representative fluorescence microscopy images of ER-ID-stained cells to evaluate endoplasmic reticulum (ER) expansion after 24h of treatment. Nuclei were counterstained with DAPI (blue). Panels include DAPI (nuclei, blue), ER-ID fluorescence (ER, red), and merge (DAPI + red). From top to bottom: untreated control cells (Ctrl); compound 20 (30 μM); Aβ(25-35) (40 μM); and co-treatment (20 + Aβ(25-35)). Scale bar: 100 μm. N ≥ 5. Magnification 20×.

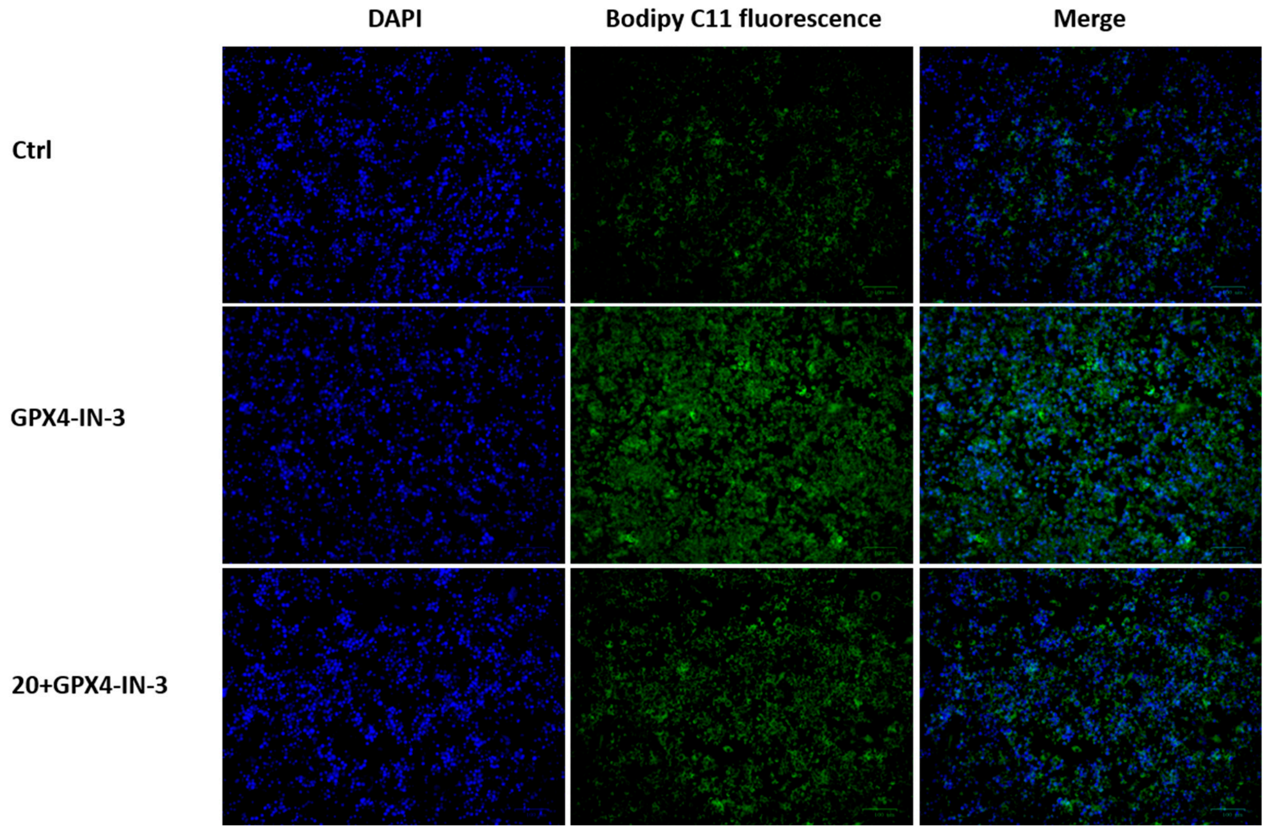

Figure S7. Representative fluorescence microscopy images of SH-SY5Y cells stained with BODIPY C11, showing lipid peroxidation after 16h of treatment after GPX4-IN-3 treatment. Nuclei were simultaneously stained with DAPI (blue). The panels include DAPI (nuclei, blue), oxidised BODIPY C11 (lipid ROS, green), and merge (DAPI + green). From top to bottom: untreated control cells (Ctrl); GPX4-IN-3 (0.5 µM); and co-treatment (20+GPX4-IN-3). Scale bar: 100 µm. N ≥ 5. Magnification 20×.
